# Reconstructing systematic persistent impacts of promotional marketing with empirical nonlinear dynamics

**DOI:** 10.1371/journal.pone.0221167

**Published:** 2019-09-18

**Authors:** Ray Huffaker, Andrew Fearne

**Affiliations:** 1 Department of Agricultural and Biological Engineering, University of Florida, Gainesville, Florida, United States of America; 2 Norwich Business School, University of East Anglia, Norwich, England, United Kingdom; University of Naples Federico II, ITALY

## Abstract

An empirical question of long-standing interest is how price promotions affect a brand’s sale shares in the fast-moving consumer-goods market. We investigated this question with concurrent promotions and sales records of specialty beer brands pooled over Tesco stores in the UK. Most brands were continuously promoted, rendering infeasible a conventional approach of establishing impact against an off-promotion sales baseline, and arguing in favor of a dynamics approach. Moreover, promotion/sales records were volatile without easily-discernable regularity. Past work conventionally attributed volatility to the impact of exogenous random shocks on stable markets, and reasoned that promotions have only an ephemeral impact on sales shares in stationary mean-reverting stochastic markets, or a persistent freely-wandering impact in nonstationary markets. We applied new empirical methods from the applied sciences to uncover an overlooked alternative: ‘systematic persistence’ in which promotional impacts evolve systematically in an endogenously-unstable market governed by deterministic-nonlinear dynamics. We reconstructed real-world market dynamics from the Tesco dataset, and detected deterministic-nonlinear market dynamics. We used reconstructed market dynamics to identify a complex network of systematic interactions between promotions and sales shares among competing brands, and quantified/characterized the dynamics of these interactions. For the majority of weeks in the study, we found that: (1) A brand’s promotions drove down own sales shares (a possibility recognized in the literature), but ‘cannibalized’ sales shares of competing brands (perhaps explaining why brands were promoted despite a negative marginal impact on own sales shares); and (2) Competitive interactions between brands owned by the same multinational brewery differed from those with outside brands. In particular, brands owned by the same brewery enjoyed a ‘mutually-beneficial’ relationship in which an incremental increase in the sales share of one marginally increased the sales share of the other. Alternatively, the sales shares of brands owned by different breweries preyed on each other’s market shares.

## Introduction

We investigate the dynamic impact of a stream of price promotions on a brand’s market share of sales in the fast-moving consumer-goods market. Price promotions include temporary price reductions, coupons, rebates, promotion packs with extra content, loyalty discounts with repeated purchases, or free goods and services contingent on the purchase of something else (e.g., a ‘two for one special’ or a free bottle of wine with dinner).

At first glance, we might well expect promotions to have a positive impact on a brand’s sales share; otherwise, why would retailers invest in them? Existing customers should increase their short-term purchases of the promoted brand in response to lower prices, and their longer-term purchases due to increased brand loyalty (from receiving benefits beyond their normal purchase) and an ‘inertia’ effect encouraging repeated purchases. New customers might be induced to switch from other brands on a trial basis [1]. However, the literature teaches us that promotions might also have a negative impact by lowering a consumer’s repurchase probability when the brand is no longer promoted [2]. Promotions could cause consumers to downgrade their perceptions of brand quality, focus too heavily on price rather than the brand’s distinguishing qualities, or lower their price expectations to the promotional level. Moreover, these positive and negative forces could operate simultaneously [1, 3]. Consequently, the ambiguous net impact of price promotions on a brand’s sales share must be resolved empirically from available promotions and sales records, with theory confirmed brick-by-brick from detected empirical regularities across diverse cases [4].

We investigate the impact of price promotions with concurrent sales and promotions time-series records of specialty beer brands pooled over all Tesco stores (the largest chain in the UK). Specialty beers are “typically regular beers brewed to a classic style (such as Porter, Stout, or Pale Ale) but with some new flavor added” http://www.dummies.com/food-drink/drinks/beer/types-of-specialty-beers/. This rich data set affords a valuable opportunity to advance empirical understanding of real-world promotional marketing dynamics. The records cover 104 weeks (February 2009 to January 2011). Weekly sales were provided by scanned purchases of Tesco ClubCard holders numbering roughly 17 million households in the UK (40% of all households) (S1 File). ClubCard purchases account for about 80% of total sales. Weekly promotions made by each brand were collected by dunnhumby—a subsidiary of Tesco focused on consumer data analysis (S2 File). As discussed more extensively below, we converted the records to mean-adjusted weekly brand shares of sales and promotions.

**Fig 1:**
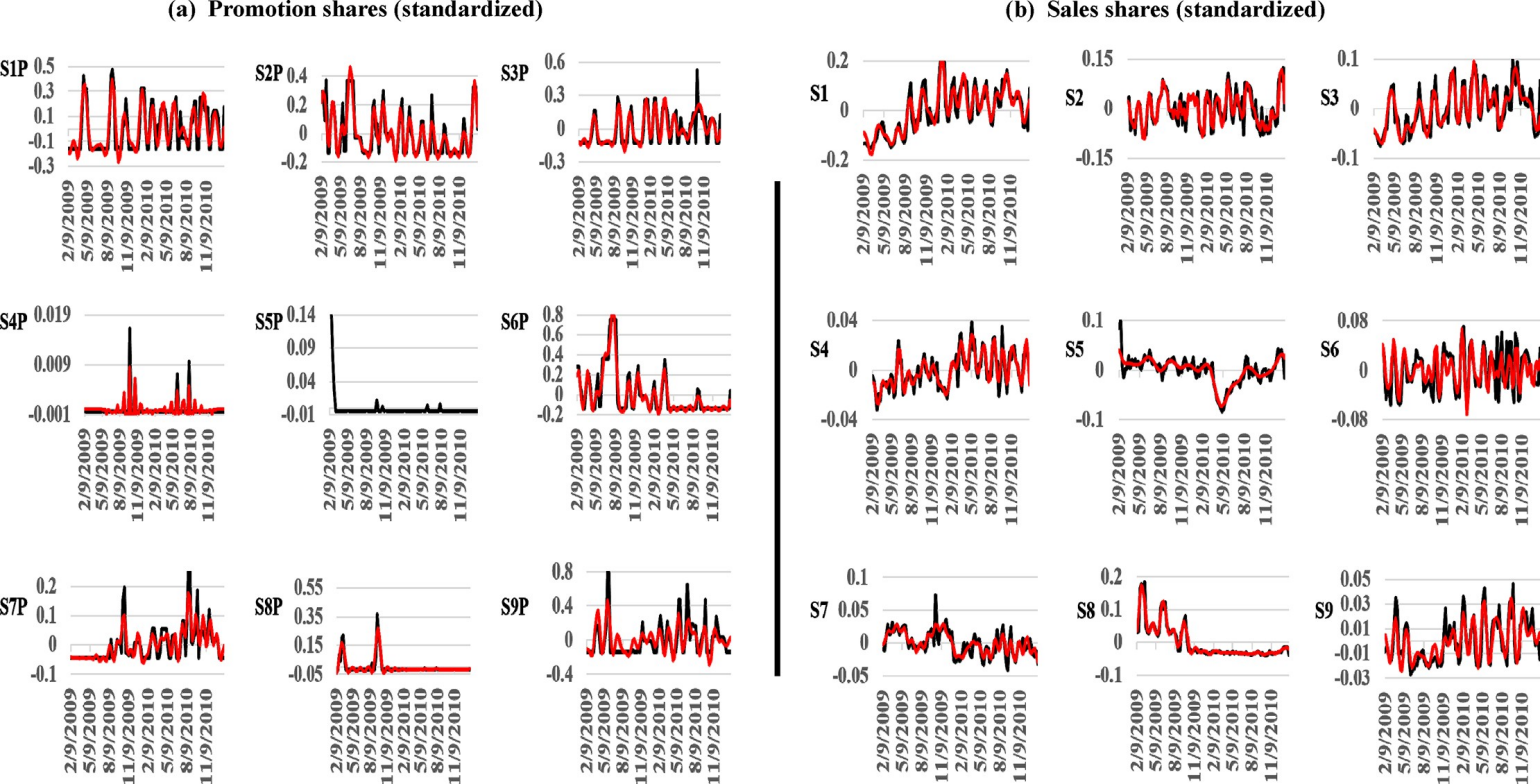
Plots of Tesco weekly promotions and sales records with isolated signals. We improve the performance of NLTS by isolating structured variation (signal) from unstructured variation (noise) in observed records (black curves) with singular spectrum analysis. Isolated signals (red curves) are dominated by slow-moving trends, a triplet of lower-frequency oscillations (13, 8.667, and 6.5 weeks), and another triplet of higher-frequency oscillations (5.2, 4.2, and 2.1 weeks).

The records are plotted in Fig 1 (black curves). We first observe that most brands were continuously promoted (Fig 1A). Past work commonly evaluated the effectiveness of isolated promotions by comparing sales after a promotion against a prior off-promotion baseline [1]. This approach works best if promotions are indeed independent events. However, when brands are continuously promoted, there are reasons to expect in theory and practice that consumer responses will be linked through time. For example, consumers may stockpile product in response to an earlier price discount, and thus be taken out of the market for a current promotion [3]. Analysts miscasting a continuous promotional stream as a sequence of independent events face the daunting—if not impossible—task of establishing an artificial baseline that cuts off the effectiveness of previous promotions without substantially biasing the results. This argues for an empirical approach that respects the dynamics of real-world promotional activity.

We next observe that the promotions and sales records are volatile without easily-discernable patterns or regularity (Fig 1A and 1B). Early work conventionally attributed observed market volatility to exogenous random shocks; and consequently, relied on an array of linear-stochastic models that restricted the range of empirically-detectable promotional marketing dynamics. In particular, these models presumed that markets self-correct as economic agents re-adjust supply to demand in response to shocks, and thus formulated sales as stationary mean-reverting stochastic processes. As a result, promotions were restricted to have only an ephemeral impact because sales would revert to pre-promotional long-term mean performance levels (see review by Dekimpe et al., 2005). Distributed-lag models (such as the ‘Koyck’ model) forcibly dampened long-term promotional impacts. These models were justified empirically on the basis that they provided good fits to the data [5].

Subsequent work worried that empirical evidence supporting ephemeral promotional impacts was more an artifact of the stationarity modeling restriction than an accurate depiction of real-world market behavior; and consequently, formulated more flexible models allowing for nonstationary sales that randomly evolve (‘freely wander’) in response to promotional shocks [6]. In particular, linear stochastic autoregressive specifications empirically distinguished between stationary and nonstationary sales by determining whether the fitted model’s autoregressive lag polynomial had a less than unitary root (stationarity), or a unit root (nonstationarity). A current change in sales was calculated as the weighted sum of promotional-marketing shocks in previous periods, where weights measured the current impact of a unit shock in a given previous period. This remains a conventional approach for detecting persistent market impacts in other applications as well [7].

In limiting market taxonomy to the dichotomy between self-correcting stationary markets and freely-wandering nonstationary markets, past work foreclosed a third possible explanation for real-world volatility: Markets may be endogenously unstable (not self-correcting) as economic agents are prevented from smoothly re-adjusting supply to demand due, for example, to financial or institutional constraints. In a series of articles entitled “Big Economic Ideas” [8], *The Economist* recommended that “like physicists, [economists] should study instability instead of assuming that economies naturally self-correct” [9]. Similarly, Galtier (2013) identified diagnosing whether markets are inherently unstable as the key to evaluating the effectiveness of various public market interventions to counter threats to food security from food-price volatility [10]. Acknowledging the possibility of endogenously unstable markets opens the door to the possibility of ‘systematic persistence’ in which the economic impacts of strategic (not random) promotions persist and evolve systematically (not freely wander) in accordance with a complex network of nonlinear state-dependent interactions encoding the history of moves and countermoves of competing brands.

Whether volatility in market records is stochastically or deterministically forced makes a profound difference in how to reliably assess the dynamic impact of promotions. In a linear-stochastic world, we look for randomly-drifting interactions among market variables. In a nonlinear-deterministic world, we look for systematic state-dependent interactions that depend mechanistically on the levels of market variables over time. Indeed, systematic interactions are implied by the mechanistic nature of empirical questions posed in a survey paper by Blattberg et al. (1995): Do a brand’s promotions substantially increase its own sales? Do brands use promotions to cannibalize the sales shares of competing brands in the battle for valuable shelf space in retail outlets?

Since neither a linearly-stochastic or nonlinear-deterministic world is compelled by theory, we let the data guide the selection. This reduces the risk that mistaken presumptions one way or the other distort assessment of real-world impacts of a stream of promotions. We take an innovative data-diagnostic approach that seeks to reconstruct real-world market dynamics directly from observed time-series records on sales and promotions. Our approach operates in the initial inductive window of the classic scientific method in which “[scientists] are presented with observations and asked to build theories…to go backward, to solve for [the system] that made them” [11]. The discipline of nonlinear dynamics has solved the backward problem of mathematically reconstructing system dynamics from system output without knowledge of underlying equations [12]. Nonlinear Time Series Analysis (NLTS) adapts these results to empirically reverse-engineer real-world system dynamics from observable output data given that the real-world system is largely unknown [13, 14]. A rich array of NLTS-based methods has recently emerged in other disciplines that leverages empirically-reconstructed dynamics to identify and measure dynamic causal interactions in real-world networks [15, 16].

A reverse-engineering approach to empirical economics contrasts with more conventional model-centric approaches that indirectly simulate market dynamics with models fitted to the data, and presume that good fits imply real-world correspondence [17, 18]. However, relying on goodness-of-fit to empirically validate models commits the logical fallacy of ‘affirming the consequent’: If A, then B; B, therefore A. In the context of model validation: If the model is true, it provides a good fit; this model provides a good fit, therefore it is true [19]. We can reason that a model providing a poor fit is incorrect, but we cannot presume the opposite because other models formulated differently also might be parameterized to fit well [20].

We use an NLTS data-diagnostic approach to measure time-dependent marginal impacts of a brand’s stream of promotions on its own sales share, and those of competitors with the Tesco dataset.

## Materials and methods

### Data preprocessing of Tesco sales and promotions records

Data access was made possible as a result of a longstanding relationship between Professor Andrew Fearne and the data providers, dunnhumby. Now in its fourtheenth year, this relationship provides Professor Fearne and his team of researchers with access to the retailer's loyalty card data via a web-portal that is managed by dunnhumby, for the purpose of conducting research.

Promotions were reported in the data set (S2 File) as savings to consumers: *S* = *P*_R_−*P*_P_, where *P*_R_ is the regular (pre-promotion) unit price and *P*_P_ is the promotional counterpart. Savings *S* were reported directly for simple price cuts. For more sophisticated promotions—such as *any two for £3*—the ‘equivalent single promotional price’ was reported (*P*_P_ = £3/2 = £1.50), along with the ‘depth of cut’ (*DC*) measuring the percentage decrease from the regular price (for example, 3.85%). We computed consumer savings *S* for these promotions indirectly by solving for the unreported regular price *P*_R_ from the definition of *DC* (S3 File):

$$DC=\frac{P_{R}-P_{P}}{P_{R}}\Rightarrow P_{R}=\frac{P_{P}}{1-DC}$$ (1)

and substituting it into the consumer savings equation:

$$S=\frac{P_{P}}{1-DC}-P_{p}=P_{p}\left(\frac{DC}{1-DC}\right)=£1.50(0.04)=£0.06$$ (2)

**Fig 2:**
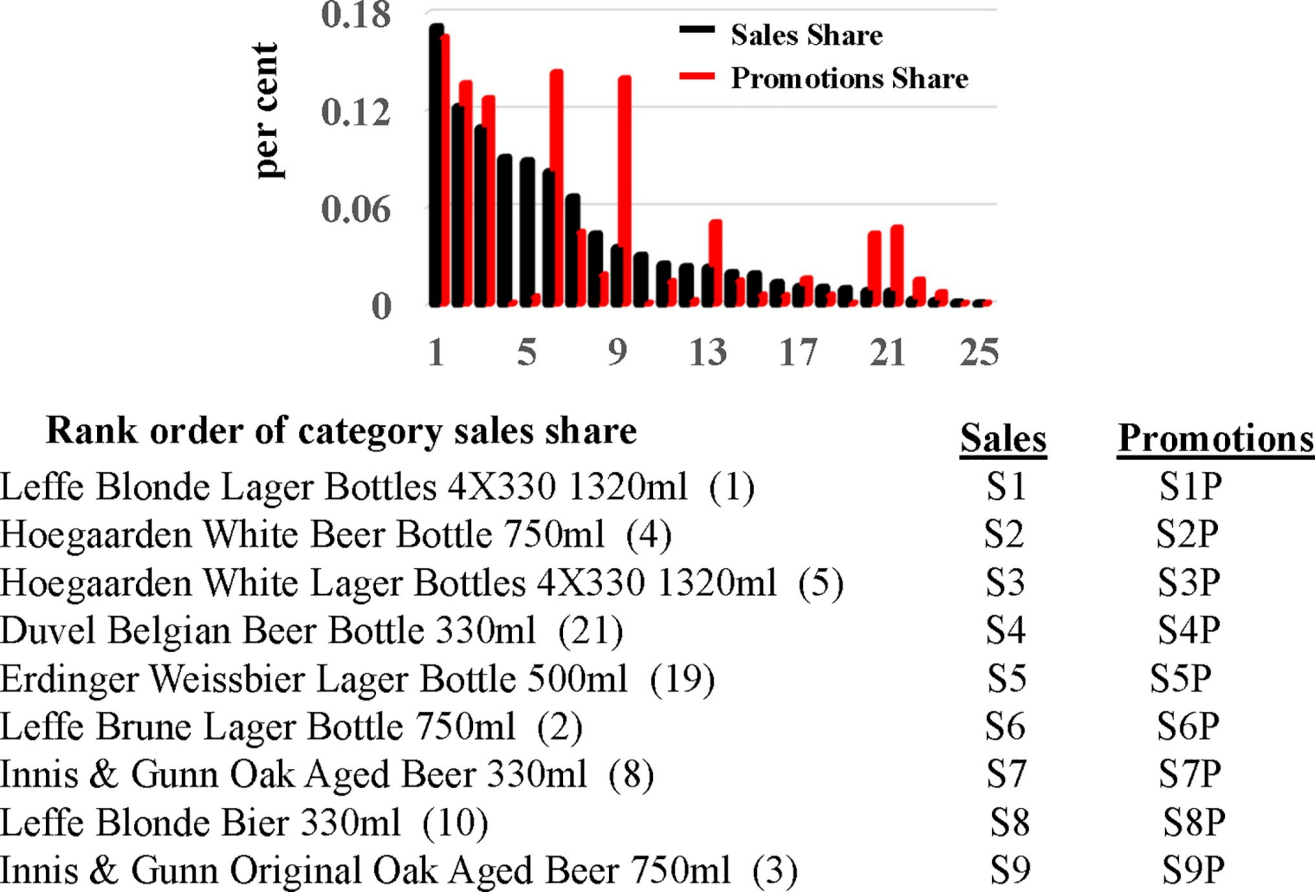
Description of Tesco dataset. The bar chart plots the category sales and promotions shares for each brand calculated over the entire 104 weeks of the dataset in declining order of sales shares. We focus on the brands with the largest nine category sales shares since they exhibited the most continuous sales/promotions records. The Leffe and Hoegaarden brands are both owned by ABInbev.

The bar chart in Fig 2 plots the category sales and promotions shares of the 25 brands competing in the specialty beer category in declining order of category sales share. We computed a brand’s category sales share as the fraction of its sales to the category total, both aggregated over the total record length (104 weeks). We computed promotions shares in the same way. We limit our investigation to brands with the top nine category sales shares since they exhibited the most continuous sales and promotions over the record length. These brands are listed in Fig 2 with the variable names used to identify them. Two of the brands (Leffe and Hoegaarden) are responsible for five of the nine packaging configurations competing in this category. For example, there are two different configurations of the Hoegaarden brand: ‘White Beer Bottle’ and White Lager Bottles’. The Leffe and Hoegaarden brands are owned by the same multinational brewery (ABInbev), and we will examine whether competition between these brands differs from their competition with outside brands in the category (e.g., Innis & Gunn).

For each of the nine retained brands, we computed weekly shares of category sales by dividing weekly brand sales by category totals for the week. We computed weekly shares of category promotions in the same way. We removed the mean (computed over the 104-week record length) from each computed sales and promotion series. These are the time-series records that we analyze below, and refer to as ‘sales shares’ and ‘promotion shares’.

### Nonlinear time series analysis (NLTS)

**Fig 3:**
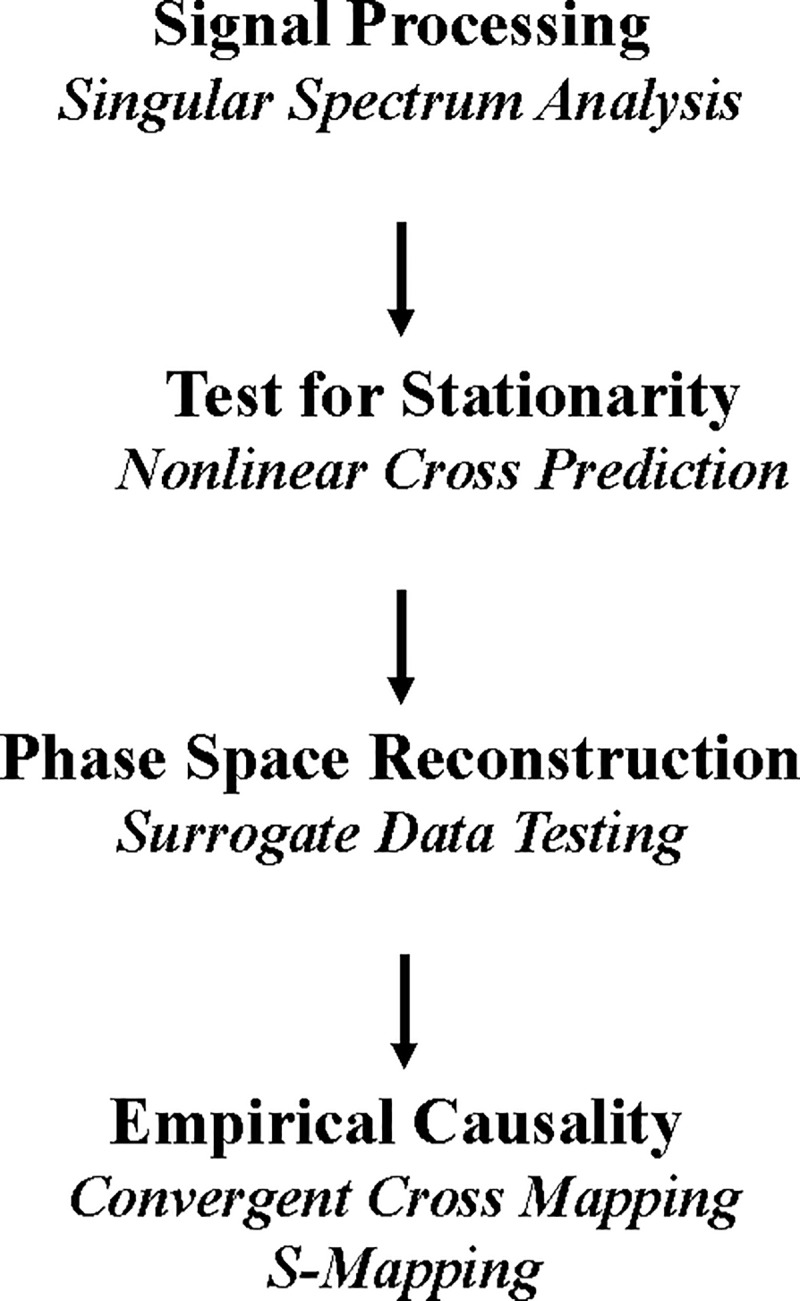
A framework for reconstructing real-world market dynamics from observed time-series records with NLTS. We implement Nonlinear Time Series Analysis (NLTS) with a sequential framework based on an extensive review of sound empirical practices recommended in the literature.

We present a sequential framework of methods for implementing NLTS (Fig 3) drawn from an extensive review of sound empirical practices recommended in the literature [14]. We introduce the methods in the order that we apply them, and in the depth facilitating discussion of results. We leave more technical descriptions to cited primary sources. We also identify the **R** packages that we used to run NLTS procedures.

#### Signal processing

The performance of NLTS depends critically on the extent to which real-world dynamics are embedded into available records, which may be noisy, short and nonstationary. The noise in records is often attributed to errors in measuring, recording, or processing data, and to random environmental shocks [21]. Consequently, we first apply the Singular Spectrum Analysis (SSA) signal processing method to isolate structured variation (‘signal’) from unstructured variation (‘noise’) in a record [14, 22, 23]. The signal can be further decomposed into a slow-moving trend component and oscillatory components. We screen for records with ‘strong’ signals—indicating that structure has greater weight in the processing than noise—and subsequently test them for deterministic nonlinear dynamics.

SSA proceeds in three stages: In the ‘decomposition’ stage, we construct a trajectory matrix whose columns are the observed record followed by its forward-delayed copies. We fix the dimension of the trajectory matrix with the ‘window length’ parameter (*L*), typically set proportionate to the dominant cycle length in the Fourier power spectrum and less than half of the record length [22, 24]. We decompose the trajectory matrix into the sum of new matrices with singular value decomposition. Each new matrix is composed of an ‘eigentriplet’, which is the product of an eigenvalue, and its corresponding right and left eigenvectors. The eigenvalue measures the weight in the decomposition attributed to the particular matrix component. In the ‘grouping’ stage, we turn to diagnostics provided by singular value decomposition to split the decomposed matrices into groups corresponding to various signal and noise components of the time series. The cumulative weights attributed to matrices grouped into the signal component provides the measure of signal strength. Finally, in the ‘reconstruction’ stage, we transform these matrix groups back into time series vectors with ‘diagonal averaging’ [22].

#### Stationarity

We next test whether a strong signal is stationary; that is, whether it exhibits similar dynamic behavior throughout its duration [25] that we can justifiably attempt to reconstruct as a single dynamic system with NLTS [26]. In particular, we search for ‘change points’—indicating time periods in which abrupt structural changes in dynamic behavior have occurred—with the Singular Spectrum Transformation (SST) approach [27, 28].

SST is implemented by applying SSA in a sliding window through the signal. We first select the ‘change-point window’ that partitions the signal into past and future series of equal length centered around a given reference time. There are no precise rules for setting the width of the non-overlapping windows. Past studies recommend running SST for wide intervals of window widths to ascertain whether results remain stable [27]. We perform SSA on the past and future series, and compute a ‘change-point score’ (CP-score) indicating the extent to which the SSA decomposition substantially changes [27]. We then increment the reference time and re-compute the CP-score. The result is a curve of CP-scores across time periods within the sliding windows.

We test the statistical significance of the CP-scores along this curve by bootstrapping an upper 95% confidence levels using randomized surrogate data vectors [27]. CP-scores falling below confidence levels are not statistically significant, and consequently do not represent structural shifts. Since we use surrogate data testing throughout the paper, we discuss it in the following section, and detail its application to CP-scores at that point.

#### Surrogate data testing

Surrogate data testing proceeds in three steps [29, 30]. First, we generate an ensemble of surrogate data vectors that destroy temporal structure in the signal, while maintaining shared statistical properties providing stochastic explanations for the behavior of a ‘discriminating statistic’ (such as the CP-score) computed from observed data. The simplest surrogate data vectors—IID (identically and independently distributed) surrogates—are constructed by shuffling the signal multiple times to destroy its serial structure. Statistically speaking, IID surrogates result from random draws without replacement from the same probability distribution as the observed time series. However, in practice, surrogates are generated to construct more complex stochastic processes. The most commonly used—amplitude-adjusted Fourier transform (AAFT) surrogates—are constructed as a linear-stochastic random variable. We generate AAFT surrogates in testing for statistical significance throughout the paper. Next, we compute the selected discriminating statistic for the observed data and each surrogate data vector.

Finally, we apply nonparametric rank order statistics to test whether the discriminating statistic computed from observed data is significantly different from the those computed from the surrogate ensemble [30]. We generate *S* = (2*k*/*α*)−1 surrogates for a two-tailed test and *S* = (*k*/*α*)−1 for a single-tailed test—where *α* is probability of a ‘false positive’ (i.e., rejecting a true null hypothesis), and *k* determines the number of surrogates with larger *k* values providing more sensitive tests. The discriminating statistic computed from the observed data is statistically significant if it falls within the extreme ranges of surrogate measurements ranked in descending order; that is, among the *k* largest (smallest) for an upper-tailed (lower-tailed) test.

We use the surrogate data method to test whether CP-scores computed from sales/promotions signals are statistically significant; and consequently, that the corresponding signals are nonstationary. We compute CP-score curves for each surrogate data vector, and rank the array of surrogate CP-scores in descending order for each time period within the sliding windows. Running an upper-tailed test, the CP-scores computed from the data are significant if they rest above an upper 95% confidence limit composed of the lowest of the *k* largest surrogate CP-scores for each time period. A signal is deemed to be nonstationary if its CP-score curve rests above the upper 95% confidence limit. NLTS requires stationary signals, so we screen nonstationary signals from further analysis.

#### Phase space reconstruction

The cornerstone of NLTS is phase space reconstruction, which is a mathematically rigorous method for reverse-engineering real-world system dynamics from observed data. Phase space is the graphical portrayal of deterministic system dynamics [31]. Phase space coordinates are provided by the system variables, and each multidimensional point records the levels (states) of system variables at a point in time. Phase space trajectories connecting these points depict the co-evolution of system variables from given initial states. If system dynamics are ‘dissipative’, these trajectories are bounded within a low-dimensional subset of phase space, and forever evolve along an ‘attractor’ in this subspace—a geometric structure with noticeable regularity [32]. Dissipative dynamics are ‘dimension reducing’—an especially useful modeling property since long-term system dynamics can be investigated with relatively few degrees of freedom regardless of the complexity or dimensionality of the system generating the data [33].

**Fig 4:**
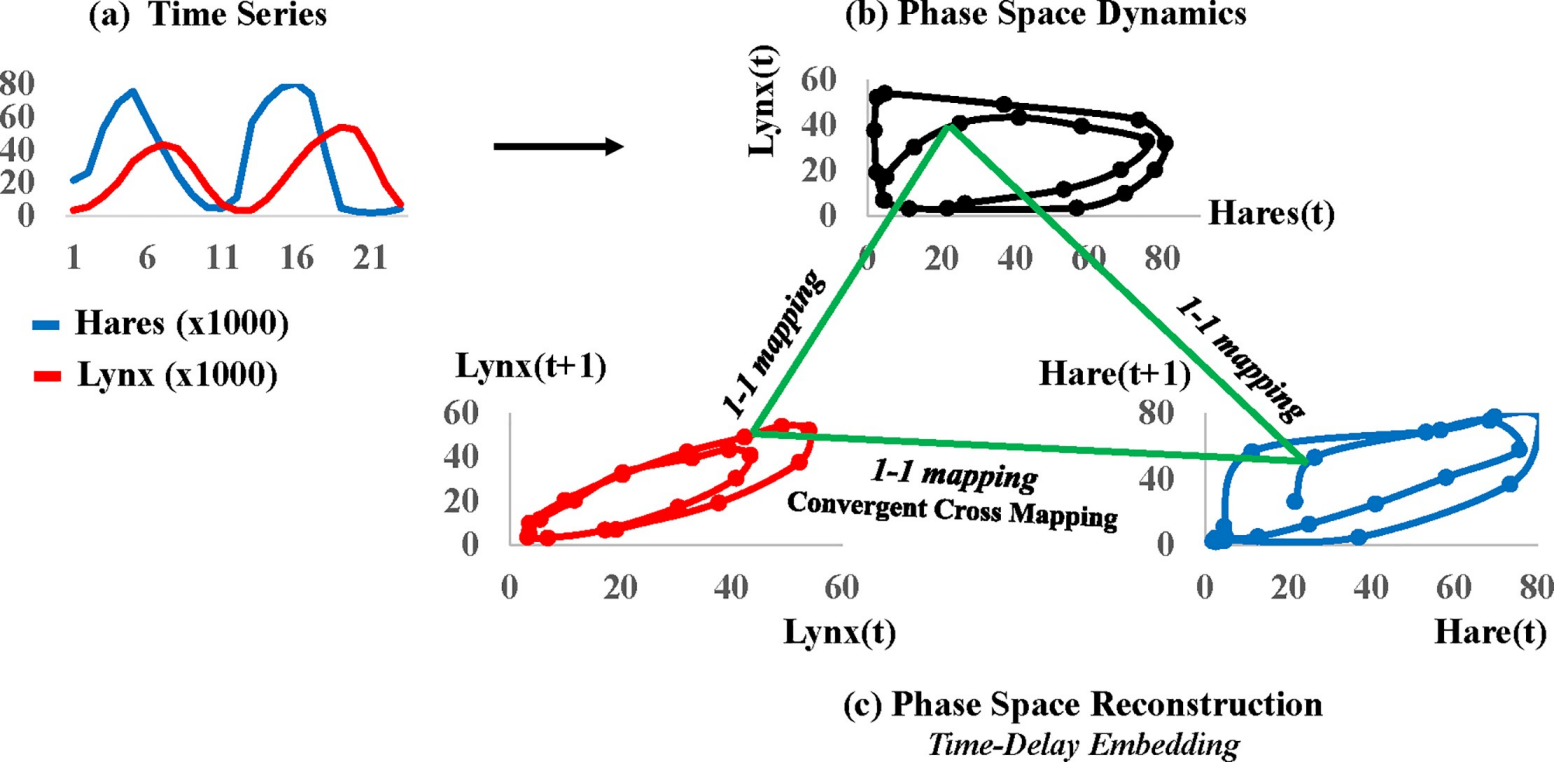
Time-delay embedding method of phase space reconstruction. **(a)** As an illustration, we reconstruct a phase space attractor from time series records on hare and lynx populations [34]; **(b)** The original phase-space attractor results from scatter plotting the observed populations at each point in time; **(c)** Using ‘time-delay embedding’, a ‘shadow’ attractor can be reconstructed from the perspective of only one of the populations by using its forward lagged copy as a surrogate for the omitted population. Original phase space dynamics map 1–1 to the reconstructed shadow phase space dynamics so long as reconstructed spaces have sufficient dimensions to contain the original attractor [12]. Since reconstructed attractors map 1–1 with the original attractor, they map 1–1 with each other. This provides the basis for the ‘convergent cross mapping’ method of detecting causality in complex dynamic systems [15].

We illustrate phase-space dynamics with data on snowshoe hare and lynx populations collected by the Hudson Bay Company in Canada from 1845 to 1935 [34]. The time-series plots show that the populations cycle through time (Fig 4A), and the phase-space portrait (obtained by plotting lynx against hares at each point in time) shows that the populations co-evolve along classic predator-prey cycles constituting the attractor for the system (Fig 4B). A large lynx population overconsumes available hares and eventually crashes for want of prey. Hares recover until pressed again by the recovering lynx population, and so on.

Phase space was limited as an empirical tool for recovering real-world dynamics from data because early practitioners assumed that they required time series records on all system variables. Obviously, one cannot hope to identify or measure all of the variables interacting in real-world dynamic systems. However, a major breakthrough in empirical dynamics occurred when mathematicians proved that phase space dynamics could be reconstructed from even a single variable by using its delayed copies as surrogates for unobserved variables [35]. An intuitive explanation is that each variable in an interdependent dynamic system encodes the history of its systematic interactions with the other variables. Returning to the Hudson Bay Company data, recall that we constructed the original predator-prey attractor using both the lynx and hare population records (Fig 4B). Now we reconstruct a *shadow* version of this attractor with only one of the variables and its one-period forward-delayed copy serving as a surrogate for the omitted variable (time-delay embedding). The shadow attractor reconstructed from only the lynx (hare) population is shown in the leftward (rightward) plot in Fig 4C. The green lines emphasize that the shadow attractors correspond one-to-one with the original attractor, and thus correspond one-to-one with each other [15].

**Fig 5:**
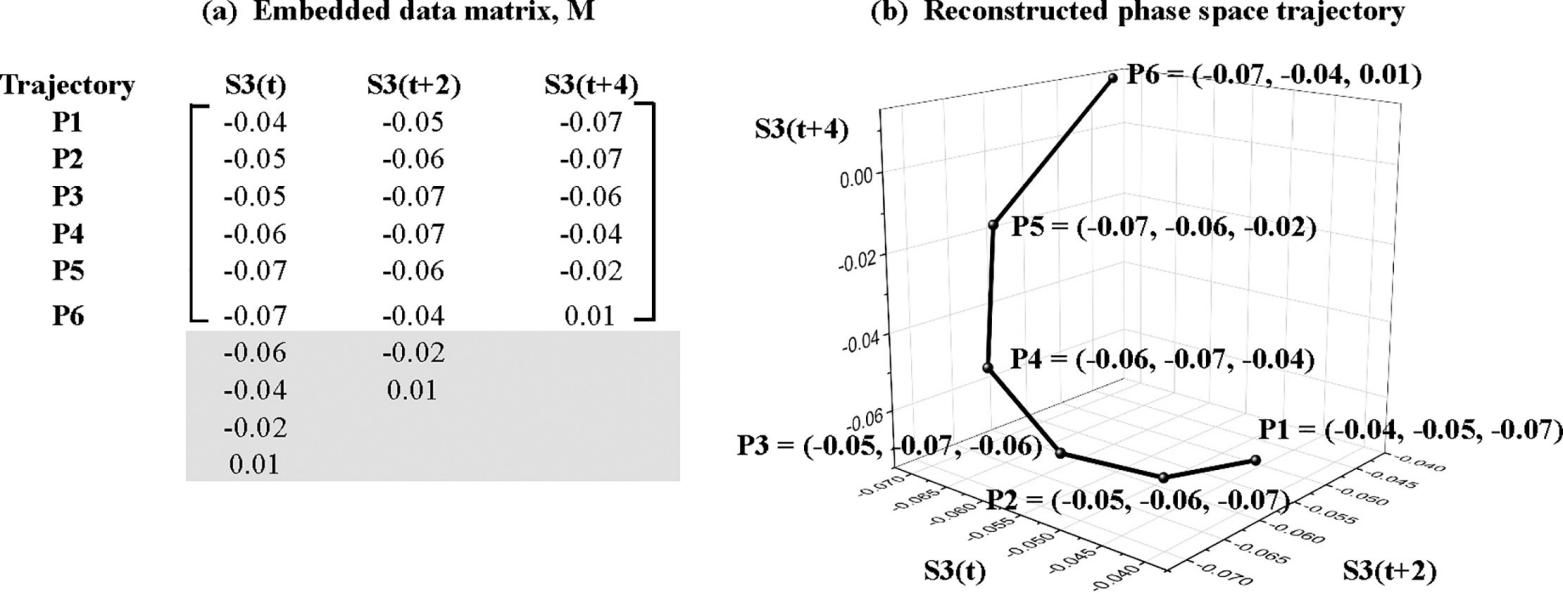
The embedded data matrix and reconstructed phase space. **(a)** The time-delay method of phase space reconstructed is implemented by constructing an ‘embedded data matrix’, *M*, with the observed time series in the first column followed by columns storing its forward-delayed copies. For example, we embed the first ten observations of the S3 record, where observations in the gray rectangle are lost to the forward-lagging process; **(b)** The scatter plotted rows of *M* are multidimensional points on a reconstructed attractor.

Takens (1980) formally proved that time-delay embedding provides a 1–1 mapping of system dynamics from the original phase space (constructed with all system variables) to the reconstructed shadow phase space so long as the latter has sufficient dimensions to contain the original attractor (at least two dimensions in the Hudson Bay Company example). In general application, we construct an *embedded data matrix*, *M*, with the observed time series in the first column followed by other columns storing its forward-delayed copies. For example, we embed the first ten observations of the S3 record in Fig 5A. In the first column of *M*, S3 is unlagged; in the second column, S3 is forward-lagged by two periods; and in the third column, S3 is forward-lagged by four periods. Observations in the gray rectangle are lost to the forward-lagging process. The rows of *M* are multidimensional points on a reconstructed attractor. For example, the first point on the attractor is P1 = (-0.04, -0.05, -0.07). The geometric representation of the reconstructed attractor is a scatterplot of all rows of *M* (Fig 5B).

The embedding process requires selection of three parameters: the ‘embedding delay’ (number of periods separating delayed copies), the ‘Theiler window’ (correction for serial correlation), and the ‘embedding dimension’ (number of columns in the embedded data matrix, *M*). The search for embedding parameters is aided by statistical rules-of-thumb designed to select values reconstructing attractors with the clearest resolution [13, 36].

The embedding delay is conventionally selected as the lag giving the first minimum of the average mutual information function—a probabilistic measure of how a time series relates to successively delayed copies of itself. This is thought to introduce a delay that is not too short for system dynamics to evolve, but not so long that it skips over important dynamic structure [36]. The Theiler window can be estimated as the lag giving the first minimum in the autocorrelation function [25]. Time series observations within the window are not used in estimating the embedding dimension because they reflect proximity in time rather than the attractor’s geometric structure [37]. Finally, once the embedding delay and Theiler window are estimated, the embedding dimension is conventionally selected with the false nearest neighbors test [36]. An attractor is initially reconstructed in two dimensions, and distances between points on the attractor are computed. This exercise is repeated in three dimensions, and the percentage of points growing farther apart from two to three dimensions (false nearest neighbors) is computed. If the percentage of false nearest neighbors is below a selected tolerance level, then two dimensions is judged to be sufficient to contain the reconstructed attractor; otherwise, the test proceeds to the next higher dimension. An attractor reconstructed in too few dimensions does not have space to fully express itself, while one constructed in too many loses geometric clarity.

In practice, we are not limited to using only one observed variable to reconstruct phase space. Takens’ theorem has been generalized to ensure that original phase space dynamics are also preserved in reconstructions using different combinations of system variables and their delayed copies [38]. This is a very useful result in empirical practice because it allows us to reconstruct a real-world attractor from multiple perspectives depending on data availability and research objective.

Surrogate data testing provides a statistical safeguard against mistaking apparent geometric regularity in a reconstructed attractor for deterministic nonlinear dynamic structure [29, 30]. We reconstruct attractors from the observed record and each surrogate data vector. AAFT surrogates (discussed above)—the most popular surrogates in practice—are generated to test the hypothesis that an empirical attractor is reconstructed from a linear-stochastic random variable.

Conventional discriminating statistics used to compare hallmark characteristics of nonlinear dynamic behavior in the observed and surrogate attractors include an attractor’s ‘fractal dimension’, the ‘maximum Lyapunov exponent’ measuring an attractor’s sensitivity to initial conditions (i.e., whether trajectories on the attractor exponentially diverge over time), an attractor’s short-term nonlinear predictive skill, and an entropy complexity measure, such as ‘permutation entropy’ [39]. We selected permutation entropy as the discriminating statistic because we could calculate it most reliability given the short duration of the time-series records in our case study. Permutation entropy modifies the classic Shannon H measure of the information contained in a time series for application to finite noisy data [40]. When *H* = 0, the time series is perfectly predictable from past values. *H* achieves a maximum value when time series observations are independent and identically distributed. Consequently, large values of *H* indicate more random behavior.

#### Convergent cross mapping

The next question is whether the attractors reconstructed from the different perspectives of multiple observed signals reconstruct the same real-world dynamic. If so, then the corresponding signals are deemed to causally interact in the same real-world dynamic system. Convergent cross mapping (CCM) was developed to answer this type of question [15]. The logic underlying CCM is that, if variables *X* and *Y* interact in the same dynamic system, then attractors reconstructed from delayed copies of *X* (*M*_*X*_) and delayed copies of *Y* (*M*_*Y*_) map 1–1 to the original attractor (*M*), and consequently map 1–1 to each other (as depicted in Fig 4B and 4C). CCM tests whether a 1–1 mapping exists between *M*_*X*_ and *M*_*Y*_ by measuring the skill with which one attractor can be used to cross-predict values on the other. For example, the notation *XxmapY* indicates that *M*_*X*_ is used to cross-predict *Y*. This asks whether *Y*’s dynamics are embedded into *X*’s long-term dynamics (*M*_*X*_); in other words, whether *Y forces X*.

**Fig 6:**
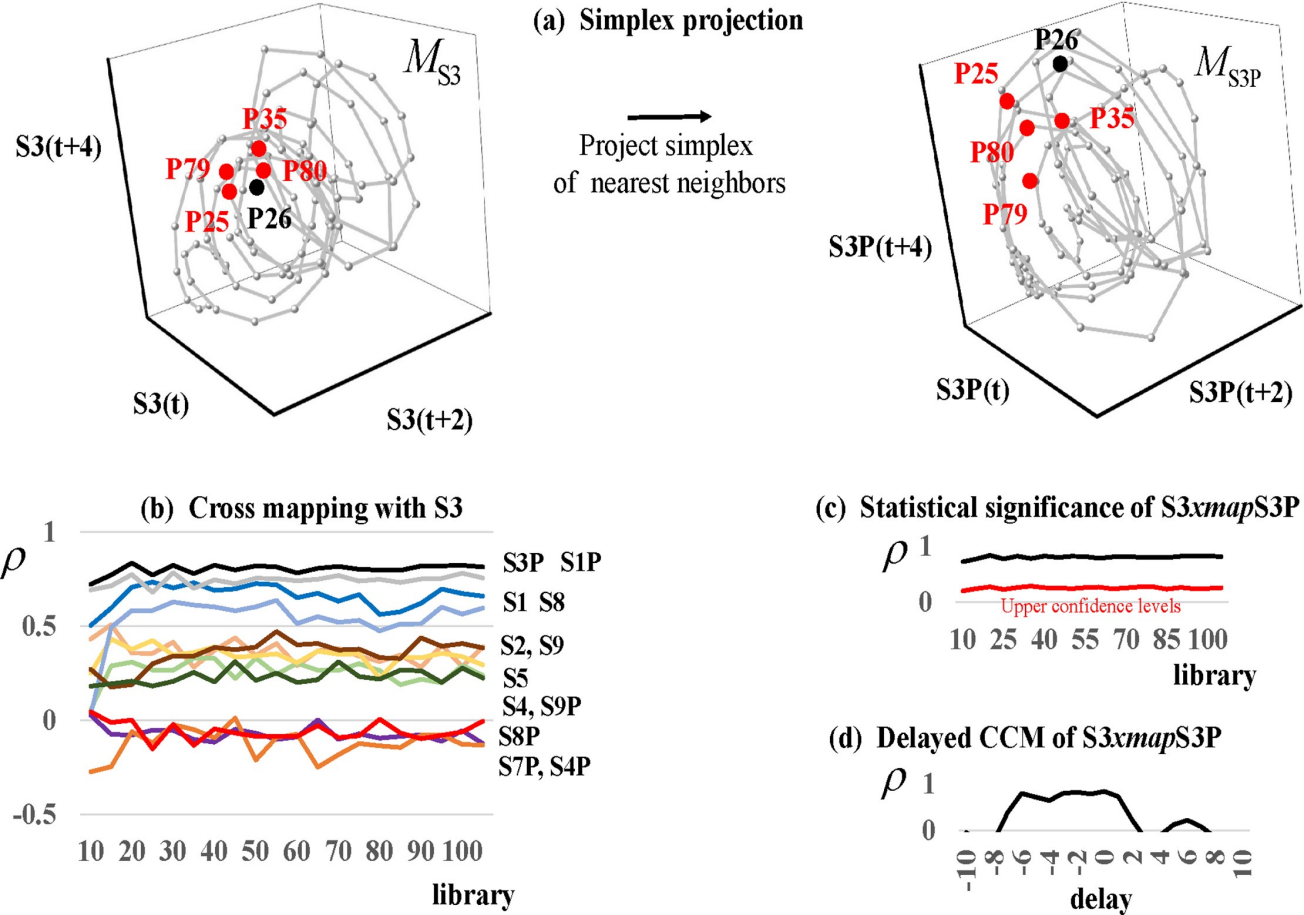
Convergent cross mapping. **(a)** Convergent Cross Mapping (CCM) operates with a ‘simplex-projection’ algorithm [15]. We illustrate this with the cross mapping S3*xmap*S3P. We reconstruct the attractors *M*_S3_ and *M*_S3P_ using S3 and S3P and two of their forward-delayed copies as phase-space coordinates, respectively, and fix a single reference point (P26) on *M*_S3_ at week 26 (left plot). We next identify the reference point’s *m*+1 nearest neighboring points, where *m* = 3 is the embedding dimension of *M*_S3_. These neighboring points (P80, P79, P35, and P25) form a *simplex* around the reference point on *M*_S3_ that is projected onto *M*_S3P_ by transferring the time coordinates of the reference point and nearest neighbors from *M*_S3_ to *M*_S3P_ (right plot). If there is a 1–1 mapping from *M*_S3_ to *M*_S3P_, then these points will be nearest neighbors on *M*_S3P_ as well, and their weighted average will skillfully predict the corresponding reference point on *M*_S3P_ (P26). This is repeated for a sampling of reference points on the *M*_S3_. Cross prediction skill is measure with the correlation coefficient between reference points on *M*_S3P_ and their predicted values; **(b)** Predictive skill should converge as the number of points used to construct *M*_S3_ (the ‘library’) increases. The figure shows the skill with which *M*_S3_ cross predicts other variables in the dataset. Convergent correlation coefficients closer to one reflect stronger causal interactions; **(c)** Statistically significant cross mappings must rest above upper 95% confidence bounds constructed with surrogate data; and **(d)** To distinguish causal interaction from synchronized behavior, cross mappings run at backward and forward delays must perform best at nonpositive delays as in the figure.

Sugihara et al. (2012) perform cross mapping with a simplex-projection algorithm. We illustrate this with the cross mapping S3*xmap*S3P in Fig 6A, where S3 is called the ‘library’ variable and S3P the *target* variable. We first reconstruct the attractors *M*_S3_ and *M*_S3P_ using S3 and S3P and two of their forward-delayed copies as phase-space coordinates, respectively. In this illustration, we fix a single reference point on *M*_S3_ at week 26 (P26). More generally, CCM is run over a sampling of points on the attractor. We next identify the *m*+1 nearest neighboring points to the reference point, where *m* = 3 is the embedding dimension of *M*_S3_. To locate nearest neighbors, we compute Euclidean distances between the multidimensional points on *M*_S3_ (i.e., between the reference point and the other rows of the corresponding embedded data matrix). In general, the Euclidean distance between two tridimensional points *P*(*p*_1_,*p*_2_,*p*_3_) and *Q*(*q*_1_,*q*_2_,*q*_3_) is:

$$\left\|P-Q\right\|=\sqrt{{\left(p_{1}-q_{1}\right)}^{2}+{\left(p_{2}-q_{2}\right)}^{2}+{\left(p_{3}-q_{3}\right)}^{2}}$$ (3)

The nearest *m*+1 = 4 neighbors to reference point P26 on *M*_S3_ are P80, P79, P35, and P25 (Fig 6A, left plot). These neighbors form a *simplex* around the reference point on *M*_S3_ that is projected onto *M*_S3P_ by transferring the time coordinates of the reference point and nearest neighbors from *M*_S3_ to *M*_S3P_ (Fig 6A, right plot). If there is a 1–1 mapping from *M*_S3_ to *M*_S3P_, then these points will be nearest neighbors on *M*_S3P_ as well.

Sugihara et al. (2012) establish this by seeing how well the reference point on *M*_S3P_ (P26) can be predicted with a locally-weighted average of the nearest neighboring points transferred from *M*_S3_:

$$\hat{P26}|M_{S3}=\sum_{i=1}^{m+1}w_{i}P_{nn}(t_{i})$$ (4)

where *P*_*nn*_(*t*_*i*_) is the nearest neighbor in the *i*^*th*^ week. The nearest neighbors closest to the reference point on *M*_S3_ (P26) receive the greatest weight (*w*_*i*_) according to:

$$w_{i}=u_{i}/\sum u_{j}\;i,j=1,\ldots ,m+1$$ (5)

where

$$u_{i}=\exp\left[\frac{-\left\|P26-P_{nn}(t_{i})\right\|}{\left\|P26-P_{nn}^{closest}\right\|}\right]$$ (6)

and $P_{nn}^{closest}$ is the closest neighboring point. The goodness-of-fit between the reference point on *M*_S3P_ (P26) and its predicted value $\hat{P26}$ can be measured with the Pearson correlation coefficient (*ρ*).

The cross-mapped predictions for a sampling of reference points on the attractor must pass three tests before being accepted as representing a real-world causal interaction. The first is a convergence test demanding that the simplex-projection prediction algorithm (Eqs 4–6) becomes more skillful as the portion of the record for S3 used to reconstruct *M*_*S3*_ (the library) increases in length; in other words, as the structural information in *M*_*S3*_ increases. This occurs if *ρ* converges closer to an acceptable level as library size increases. As an example, we show convergence curves derived by using S3 (i.e., *M*_*S3*_) to cross map the other variables testing positive for nonlinear dynamics (Fig 6B). Causal interactions are stronger the further the curves are away from the origin. We see that S3 is driven most strongly by its own weekly promotions share (S3P), providing preliminary evidence that own promotions have a sustained impact. We also see that S3 is driven by the weekly sales and promotions shares of competing brands (S1 and S1P, respectively). If, for example, we set a convergence threshold of *ρ* = 0.33 for the cross-mapping curves in Fig 6B, we conclude that S3P, S1P, S1, S8, S2, and S9 pass the test; while S5, S4, S9P, S8P, S7P, and S4P fail.

Second, we test each cross mapping for statistical significance. In particular, we test the null hypothesis that a cross mapping S3*xmap*S3P, for example, cross predicts with more skill than when S3 (the library variable) is replaced with surrogate library vectors. We run the cross mapping for each of *S* = (*k*/*α*)−1 AAFT library surrogates, and in an upper-tailed test, reject the null hypothesis only if S3 cross predicts with a larger *ρ* than the *k*^th^ largest surrogate *ρ* for each library in the convergence plot. In Fig 6C, we observe that S3*xmap*S3P is statistically significant since the convergence plot (black curve) rests above the upper 95% confidence curve (red curve) for each library.

Finally, we follow methods developed by Ye *et al*. (2015) to further screen statistically significant cross mappings for false positives in which synchronized behavior is confused for causal interaction. Non-interactive variables may falsely appear to interact when they are synchronized to the same external variable, for example, when two unrelated environment variables are synchronized to identical climatic or seasonal forces. In Fig 6D, we show the results of running the cross mapping S3*xmap*S3P (using the entire time series for S3 as the library) over a spectrum of negative and positive delayed responses between the driving variable (S3P) and the response variable (S3) to identify the delay for which CCM performs best. Ye *et al*. (2015) show that CCM performs best for a non-positive delay in the true causal direction. The cross mapping passes this test since CCM performs best at a zero delay, denoting instantaneous interaction between the two variables.

#### Characterizing the nature of interactions with the S-mapping method

We use the S-Mapping method [16] to measure the strength of, and characterize, interactions identified with CCM: Are detected interactions mutually-beneficial (symbiotic), mutually-detrimental (competitive), cannibalistic (predator-prey); and how do these interactions vary with time?

The S-mapping method computes a matrix of interactions among state variables as a dynamic system evolves along a phase space attractor. Similar to CCM, it operates with a simplex-projection algorithm. In this case, all the points on the attractor are projected forward one period except for the reference point, and the simplex is fitted with a locally-weighted multivariate linear regression scheme. As this is repeated for successive reference points along the attractor, the fitted lines collectively begin to map out the curvature of phase space, and the estimated regression coefficients measure slopes in the direction of each coordinate variable at each point [41]. When the S-mapping procedure is completed, we have a matrix whose rows are interactions measuring the marginal response of a selected response variable to incremental changes in itself and the other variables at each point on the attractor. The columns of this interaction matrix give the time series of each interaction. For example, if we construct an attractor using S1, S3P, and S3 as phase-space coordinates; select S3 as the response variable; define the library as the 50^th^ through 70^th^ points on the attractor; then the format of the interaction matrix is:

$$\left[\begin{array}{ccc} \frac{\partial S3}{\partial S1}(P51) & \frac{\partial S3}{\partial S3P}(P51) & \frac{\partial S3}{\partial S3}(P51)\\ \frac{\partial S3}{\partial S1}(P52) & \frac{\partial S3}{\partial S3P}(P52) & \frac{\partial S3}{\partial S3}(P52)\\ \ldots & \ldots & \ldots \\ \ldots & \ldots & \ldots \\ \frac{\partial S3}{\partial S1}(P70) & \frac{\partial S3}{\partial S3P}(P70) & \frac{\partial S3}{\partial S3}(P70) \end{array}\right]$$ (7)

**Fig 7:**
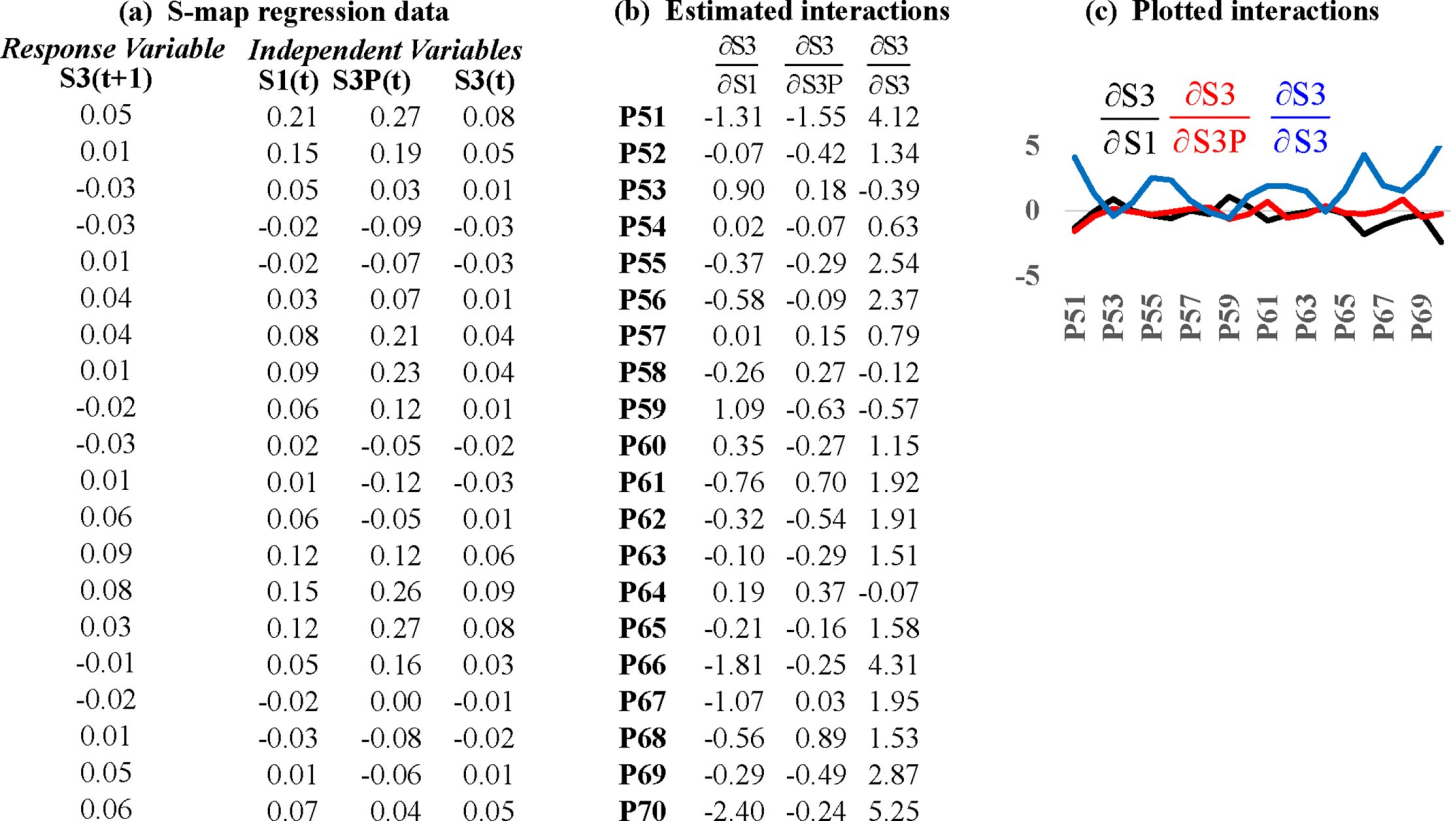
The S-mapping method of quantifying causal interactions. The S-mapping method [16] relies on a locally-weighted multivariate linear regression scheme to map out the curvature of a reconstructed attractor. This is used to compute directional derivatives that quantify marginal changes in a selected response variable to incremental changes in the others. In this example, we reconstruct an attractor with signals for S1, S3, and S3P, and take S3 as the response variable. **(a)** These signals are inputted into the locally-weighted regression scheme at a given reference point (struck out in gray); **(b)** Columns of this matrix are estimated regression coefficients and measure the interactions between S3 and the other variables; and **(c)** The interactions are plotted through time.

The data inputted into the locally-weighted regression scheme that generates Eq 7 are shown in Fig 7A. The dependent variable is the response variable advanced one period, S3(t+1), with the row of data corresponding to the reference point (P52 in this example) deleted. The independent variables are the multidimensional points on the attractor manifold. These data are imported into a weighted linear regression in which observations corresponding to nearer neigboring points are weighted more heavily according to:

$$w_{k}=\exp\frac{-\theta \left\|p_{k}-p^{ref}\right\|}{\bar{d}},\;\bar{d}=\frac{1}{n}\sum_{k=1}^{n}\left\|p_{k}-p^{ref}\right\|$$ (8)

where there are *k* = 1,…, *n* points on the attractor receiving weights (reference point *p*^*ref*^ is omitted), and *p*_*k*_ is the *k*^th^ point on the attractor. The parameter *θ*≥0 is set by the user, and determines how strongly the regression is weighted to the localized neighborhood around the reference point. If *θ* = 0, the regression reduces to a VAR model with constant coefficients, so that location does not matter on the attractor. Consequently, if *θ* is set too small, the temporal variability of interactions is biased downward. Large values of *θ* give greater weight to nearby points, making the S-map more sensitive to observational error. Supplementary material to Deyle et al. (2015) discuss strategies for setting *θ*. Fig 7B shows the matrix of interactions calculated for this example (*θ* = 10). The rows are interactions across variables for each point on the attractor. The columns are the time series for each interaction, which are plotted in Fig 7C.

#### Computational packages

We used **R** 3.4.1 and the following packages to run NLTS procedures in this paper: *Rssa 0*.*13–1* (singular spectrum analysis) [42]*; tseriesChaos 0*.*1–13* (phase space reconstruction and surrogate data analysis) [43]; *fractal 2*.*0–1* (compute AAFT surrogates) [44]; *fields 9*.*6* (compute distance matrix) [45]; *igraph 1*.*0*.*1* (plot network diagrams) [46]; *rEDM 0*.*6*.*9* (convergent cross mapping) [47]. The code used to compute interaction coefficients with S-maps can be downloaded from supplementary materials to [16]. Remaining code is detailed in [14], and can be downloaded from http://www.dista.unibo.it/~bittelli/. We used Origin 2018b graphics software for three-dimensional plotting [48].

## Results

### Signal processing

We plot signals isolated from the sales and promotions time-series records in Fig 1 (red curves). While the plotted signals visually reproduce much of the structural variation in several of the corresponding records, the objective is not to obtain a perfect match (since observed records contain noise). All of the isolated signals show strong overall strength (exceeding at least 50 percent), except for promotions of Erdinger Weissbier Lager Bottle 500ml (S5P), which we delete from further NLTS analysis (Table 1). Overall, the retained signals are dominated by slow-moving trends, a triplet of lower-frequency oscillations (13, 8.667, and 6.5 weeks), and another triplet of higher-frequency oscillations (5.2, 4.2, and 2.1 weeks).

**Table 1:** Signal processing of weekly category sales and promotions shares.

		Cycle Lengths Weeks							
Sales Records	ID	Signal Strength	Trend	2.1	4.2	5.2	6.5	8.7	13
Leffe Blonde Lager Bottles 1320 ml	S1	87%	53%				15%	8%	11%
Hoegaarden White Beer Bottle 750ml	S2	80%	24%	5%		8%	20%	12%	11%
Hoegaarden White Lager Bottles 1320ml	S3	82%					11%	16%	55%
Duvel Belgian Beer Bottle 330ml	S4	81%	32%			17%	32%		
Erdinger Weissbier Lager Bottle 500ml	S5	73%	62%						11%
Leffe Brune Lager Bottle 750ml	S6	58%			10%	23%	25%		
Innis & Gunn Oak Aged Beer 330ml	S7	64%	50%			14%			
Leffe Blonde Bier 330ml	S8	95%	56%				9%	4%	26%
Innis & Gunn Original Oak Aged Beer 750ml	S9	72%	20%				31%		21%
**Promotions Records**									
Leffe Blonde Lager Bottles 1320 ml	S1P	76%					22%	26%	28%
Hoegaarden White Beer Bottle 750ml	S2P	82%	39%			22%	21%		
Hoegaarden White Lager Bottles 1320ml	S3P	76%	15%				32%	18%	11%
Duvel Belgian Beer Bottle 330ml	S4P	56%	6%	13%	15%	22%			
Erdinger Weissbier Lager Bottle 500ml	S5P	**Deleted from NLTS due to weak signal**							
Leffe Brune Lager Bottle 750ml	S6P	90%	65%				8%		17%
Innis & Gunn Oak Aged Beer 330ml	S7P	70%	43%			16%			11%
Leffe Blonde Bier 330ml	S8P	86%	30%				9%	23%	24%
Innis & Gunn Original Oak Aged Beer 750ml	S9P	56%	16%				21%	19%	

### Stationarity testing

**Fig 8:**
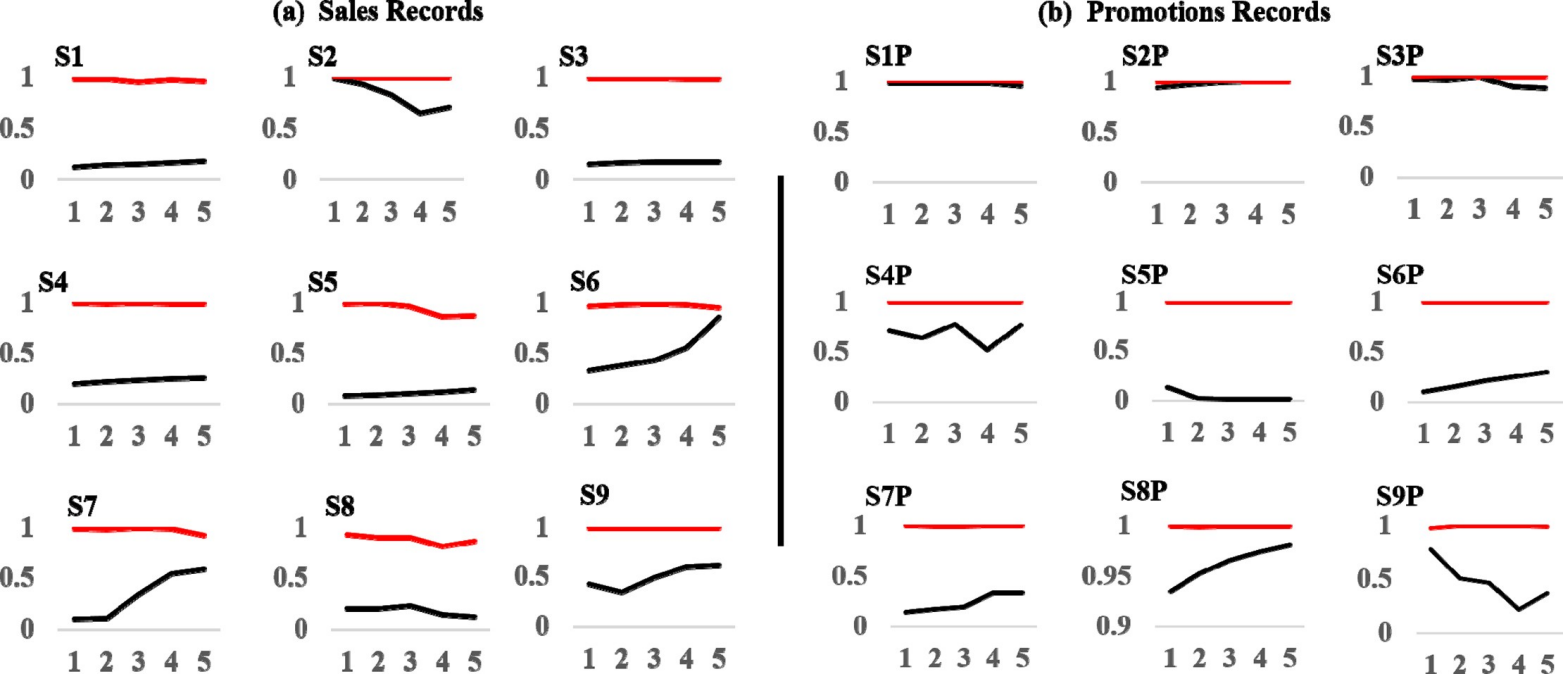
Results of nonlinear stationarity testing. We apply singular spectrum transformation [27] to test signals for stationarity required by NLTS. Change-point scores (black curves) rest below upper point-by-point 95% limits (red curves) constructed with surrogate data indicating for **(a)** sales records and **(b)** promotion records except for S2P. This indicates that S2P is nonstationary due to a significant change point in its dynamic structure, and we delete it from further NLTS diagnostics.

In applying SST to test for stationarity in strong sales and promotions signals, we set window width to 50 weeks (i.e., 25 weeks on either side of a reference point), which allowed for stable signal processing. Given that the length of the signals is 104 weeks, this created five windows. We constructed upper point-by-point 95% limits with 99 AAFT surrogate data vectors (*k* = 5). The change-point score plots (black curves) visually rest well below the upper confidence levels (red curves) for all sales signals except for S2, and all promotions signals except for S1P, S2P, and S3P (Fig 8). Of these exceptions, only the change-point score for S2P was computed to numerically exceed the confidence level. Consequently, we deem S2P to be nonstationary, and do not analyze it for deterministic nonlinear structure.

### Phase space reconstruction

**Fig 9:**
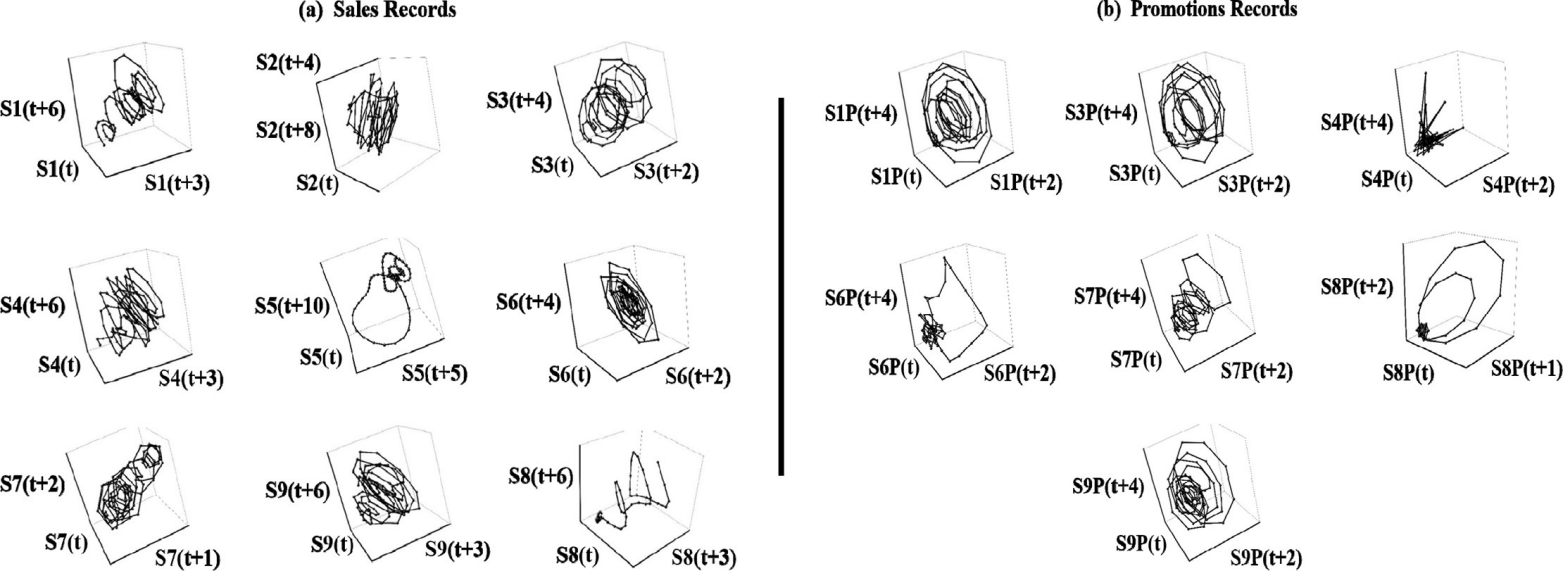
Attractors reconstructed from Tesco dataset. We successfully reconstructed low-dimensional shadow attractors from the perspectives of stationary **(a)** sale and **(b)** promotion signals. Several of the attractors exhibit striking geometric regularity characterized by oscillatory behavior detected in signal processing.

The estimated embedding parameters used to reconstruct phase space attractors from the stationary sales and promotions signals in the Tesco dataset are reported in Table 2. Most of the embedding dimensions range from two to four, with only one (S4P) reaching six. This is our first indication that the dynamics driving these signals might be low-dimensional. In Fig 9, we reconstruct a potential real-world attractor from the perspective of each signal. Several of these perspectives exhibit strong geometric regularity with a combination of low and high frequency oscillations detected in signal processing—another indication of deterministic nonlinear dynamics that we test with surrogate data.

**Table 2:** Embedding parameters.

Sales Records	ID	delay^a^	dimension^b^
Leffe Blonde Lager Bottles 1320 ml	S1	3	3
Hoegaarden White Beer Bottle 750ml	S2	4	3
Hoegaarden White Lager Bottles 1320ml	S3	2	3
Duvel Belgian Beer Bottle 330ml	S4	3	3
Erdinger Weissbier Lager Bottle 500ml	S5	5	2
Leffe Brune Lager Bottle 750ml	S6	2	3
Innis & Gunn Oak Aged Beer 330ml	S7	1	3
Leffe Blonde Bier 330ml	S8	3	3
Innis & Gunn Original Oak Aged Beer 750ml	S9	3	3
**Promotions Records**			
Leffe Blonde Lager Bottles 1320 ml	S1P	2	4
Hoegaarden White Beer Bottle 750ml	S2P		
Hoegaarden White Lager Bottles 1320ml	S3P	2	4
Duvel Belgian Beer Bottle 330ml	S4P	2	6
Leffe Brune Lager Bottle 750ml	S6P	4	3
Innis & Gunn Oak Aged Beer 330ml	S7P	2	2
Leffe Blonde Bier 330ml	S8P	1	2
Innis & Gunn Original Oak Aged Beer 750ml	S9P	2	4

^**a**^ Embedding delay estimated with mutual information function

^b^ Embedding dimension estimated with false-nearest-neighbors test. Shaded rows indicate records for which these statistics could not be calculated. These records were deleted from further analysis.

We tested the null hypothesis that visual regularity in each reconstructed attractor in Fig 9 is more likely fortuitously mimicked by linear-stochastic dynamics, with the alternative being that untested dynamic structures (such as nonlinear deterministic dynamics) remain possibilities. We ran a lower-tailed test with 199 AAFT surrogates and an *α* = 0.05 significance level to reject the null hypothesis only if permutation entropy computed from the signal falls within the lower extreme surrogate values.

We summarize the results of surrogate data testing in Table 3. The first column shows the signals tested, the second column its identification index, the third column the permutation entropy measured from the corresponding reconstructed attractor, and the fourth column the permutation entropy above which the null hypothesis of linear-stochastic dynamics is accepted. The fifth column indicates whether the null hypothesis was accepted. We accept the null hypothesis for the attractors reconstructed from the S4, S6, and S7 sales signals; and the S4P, S6P, S7P, and S9P promotions signals. These attractors were most likely generated by linear-stochastic dynamics, and we screen the corresponding signals from further NLTS diagnostics. Alternatively, we reject the null hypothesis for attractors reconstructed from the S1, S2, S3, S5, S8, and S9 sales signals, and the S1P, S3P, and S8P promotions signals. We cannot rule out the possibility that these signals were generated by real-world deterministic nonlinear dynamics.

**Table 3:** Surrogate data testing.

		Discriminating Statistic		
Sales Records	ID	Entropy^a^	Surr(low)^b^	H0^c^
Leffe Blonde Lager Bottles 1320 ml	S1	0.789	0.803	reject
Hoegaarden White Beer Bottle 750ml	S2	0.85	0.86	reject
Hoegaarden White Lager Bottles 1320ml	S3	0.79	0.8	reject
Duvel Belgian Beer Bottle 330ml	S4	0.84	0.84	accept
Erdinger Weissbier Lager Bottle 500ml	S5	0.66	0.74	reject
Leffe Brune Lager Bottle 750ml	S6	0.9	0.87	accept
Innis & Gunn Oak Aged Beer 330ml	S7	0.87	0.85	accept
Leffe Blonde Bier 330ml	S8	0.63	75	reject
Innis & Gunn Original Oak Aged Beer 750ml	S9	0.81	0.82	reject
**Promotions Records**				
Leffe Blonde Lager Bottles 1320 ml	S1P	0.79	0.81	reject
Hoegaarden White Lager Bottles 1320ml	S3P	0.78	0.81	reject
Duvel Belgian Beer Bottle 330ml	S4P	0.9	0.9	accept
Leffe Brune Lager Bottle 750ml	S6P	0.86	0.85	accept
Innis & Gunn Oak Aged Beer 330ml	S7P	0.85	0.85	accept
Leffe Blonde Bier 330ml	S8P	0.83	0.88	reject
Innis & Gunn Original Oak Aged Beer 750ml	S9P	0.81	0.81	accept

^**a**^ Permutation entropy taken from the empirically-reconstructed attractor for a record

^b^ The lower bound on entropies measured for 199 AAFT surrogates (*α* = 0.05)

^**c**^ If the entropy measurement for the empirically-reconstructed attractor does not fall below the surrogate lower bound, we accept the null hypothesis of linear stochastic dynamics. Otherwise, untested dynamic structures (such as nonlinear deterministic dynamics) remain possibilities. We do not attempt to reconstruct nonlinear dynamics from signals for which the null hypothesis is accepted, and delete them from further analysis.

### Convergent cross mapping

**Fig 10:**
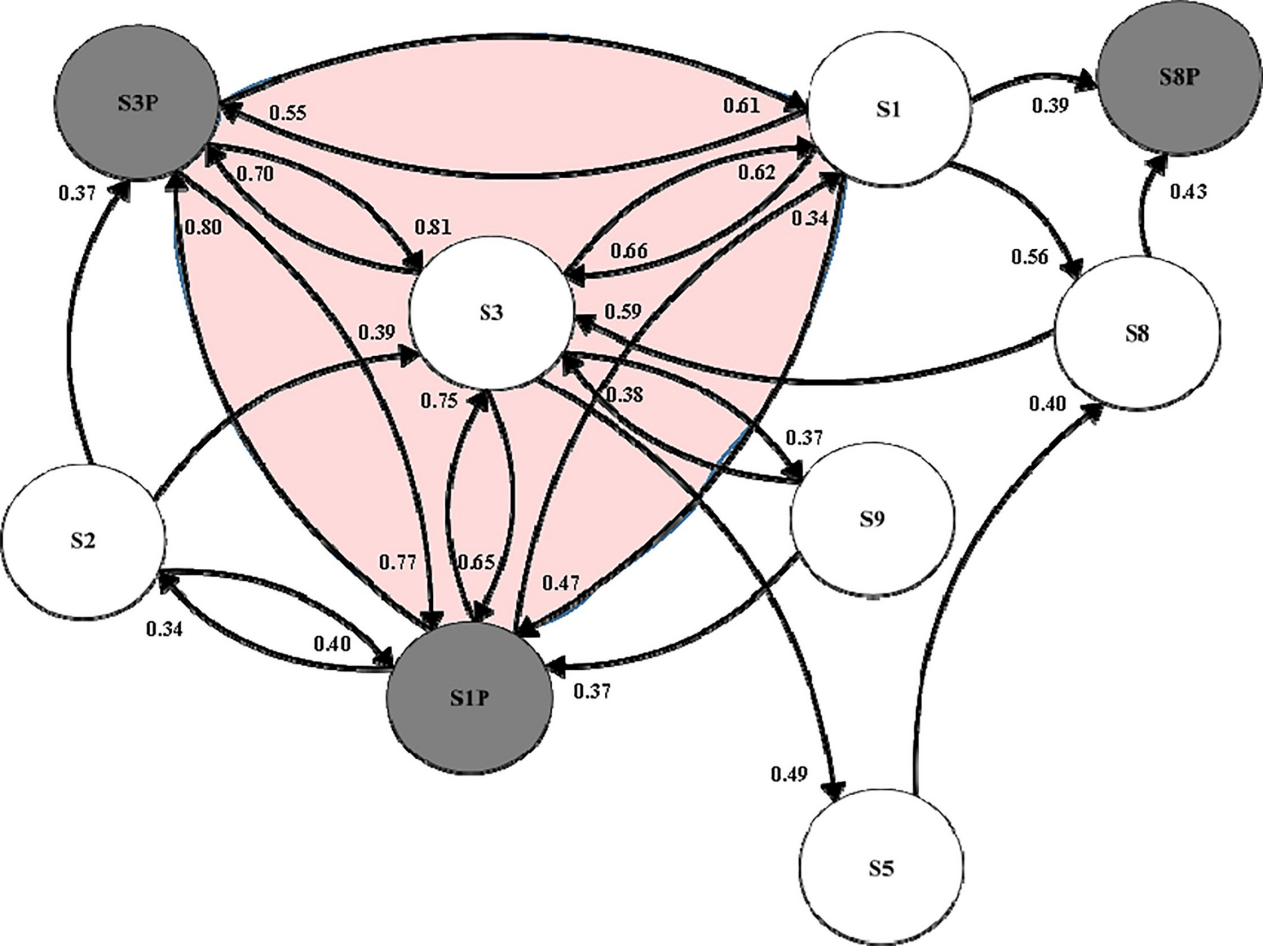
Community interaction diagram. We summarize causal interactions detected with CCM in a community interaction diagram whose nodes are sales and promotions signals screened for stationarity and nonlinear deterministic dynamic behavior. Arrows between nodes indicate the direction of interaction. The strength of the interaction is given by the fractions near each arrow head, which are the convergent correlation coefficients for each cross mapping. We focus on the area of the diagram (shaded red) containing bilateral interactions between ABInbev brand configurations Leffe Blonde Lager Bottles 4X330 1320ml and Hoegaarden White Lager Bottles 4X330 1320ml; and between these ABInbev configurations and the Innis & Gunn Original Oak Aged Beer 750ml configuration, which is not owned by ABInbev.

**Fig 11:**
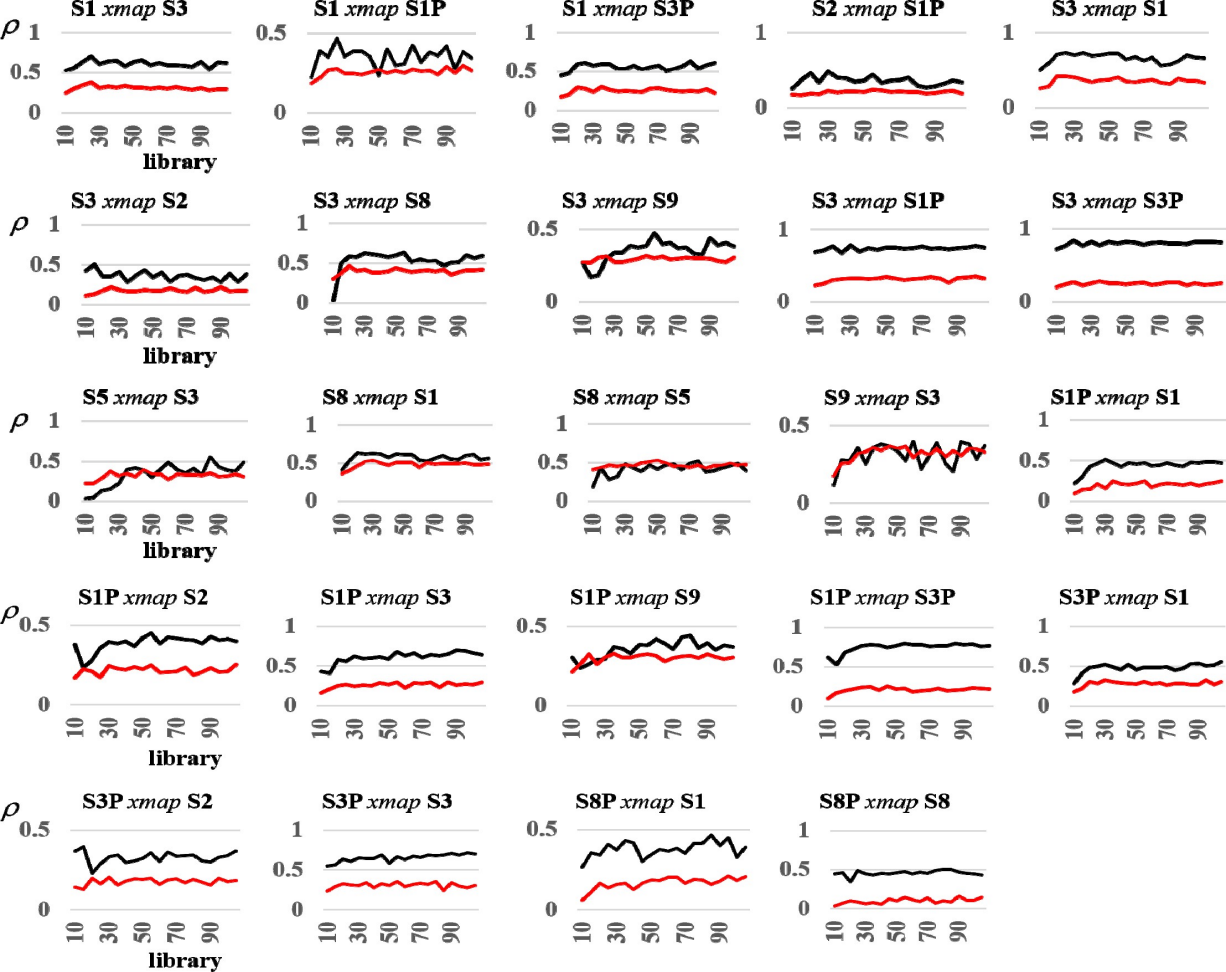
Convergent cross mapping (CCM) results. This figure reports CCM results underlying the causal interactions summarized in the community interaction diagram (Fig 10). We include interactions in the diagram associated with CCM curves (black curves) exhibiting convergent correlations exceeding 0.33 (the strength of interactions increases as convergent correlations approach 1), and resting at or above 95% confidence levels (red curves).

**Fig 12:**
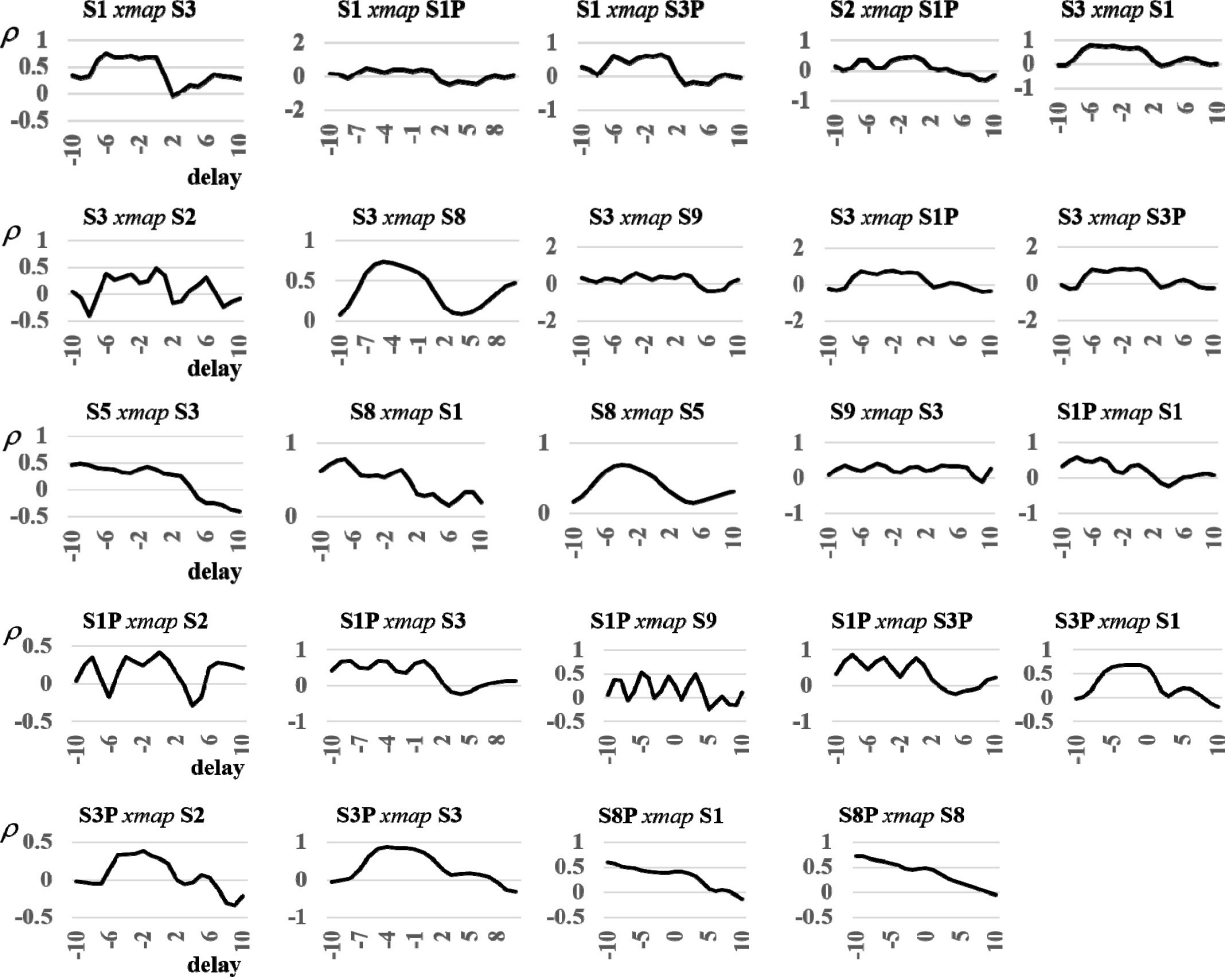
Delayed (Extended) CCM results. We screened for interactions whose cross mappings pass delayed (extended) CCM tests to rule out non-causal synchronous behavior, as demonstrated by delayed CCM curves with peaks at nonpositive delays in the figure.

We can visualize the causal interactions detected with convergent cross mapping (CCM) in a ‘community interaction diagram’ (Fig 10). The nodes represent sales and promotions signals screened for stationarity and nonlinear deterministic dynamic behavior. Arrows between nodes indicate that the signals interact in the same reconstructed real-world market system, and the direction of interaction. The strength of the interaction is given by the fractions near each arrow head, which are the convergent correlation coefficients for each cross mapping (Fig 11, black curves). For example, the convergent correlation coefficient is 0.81 for the cross mapping S3*xmap*S3P, which indicates that S3P drives S3. We include interactions in the diagram associated with CCM curves exhibiting convergent correlations exceeding 0.33 (the strength of interactions increases as convergent correlations approach 1), and resting at or above 95% confidence levels (Fig 11, red curves). In addition, we include interactions whose cross mappings pass delayed (extended) CCM tests to rule out non-causal synchronous behavior, as demonstrated by delayed CCM curves with peaks at nonpositive delays (Fig 12).

The community interaction diagram (Fig 10) begins to shed valuable light on empirical questions raised in the literature outlined above: Do a brand’s promotions substantially increase sales? Do brands use promotions to cannibalize the sales shares of competing brands? Given the nature of the Tesco dataset, we additionally consider whether this behavior changes between brands owned by different multinational breweries. A prerequisite for responding to these questions is that the underlying interactions are detected in the first place, and the community interaction diagram provides empirical evidence of this. We focus on the area of the diagram (shaded red) containing bilateral interactions between the 1^st^ (Leffe Blonde Lager Bottles 4X330 1320ml) and 3^rd^ (Hoegaarden White Lager Bottles 4X330 1320ml) leading brand configurations in category sales shares. Both brands are owned by ABInbev. The red-shaded area also includes interactions between these ABInbev brands and the 9^th^ largest sales-share brand (Innis & Gunn Original Oak Aged Beer 750ml), which is not owned by ABInbev.

### Quantifying causal interactions with S-mapping

**Fig 13:**
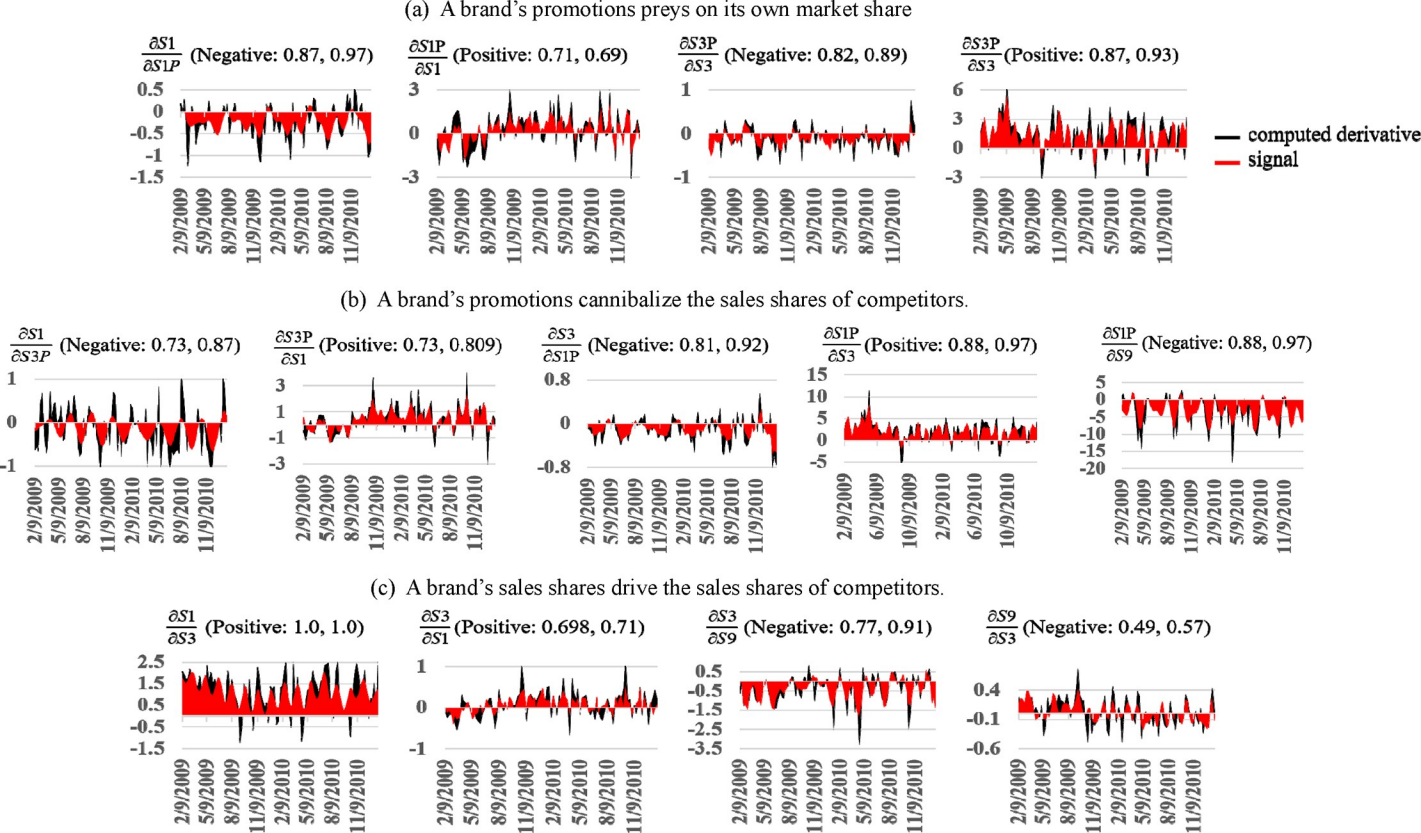
Quantified interactions. We embedded an empirical attractor with phase space coordinates provided by S1, S1P, S3, S3P, and S9, and applied S-mapping to compute partial derivatives quantifying interactions among these variables over time (black curves). The areas between the curves and the zero-axis are shaded black to highlight weeks when interactions are positive or negative. We next isolated the systematic components (signals) of the computed derivatives with singular spectrum analysis (shaded red). Since partial-derivative signals are mostly either positive or negative for the majority of weeks in the POR, we characterize them by: (1) the percentage of weeks that each partial-derivative signal is mostly positive/negative; and (2) the relative magnitude of positive/negative areas to total area between the curve and the zero-axis. These measures are reported in the headings to each plot. For example, '∂S1/∂S1P (Negative: 0.87, 0.97)’ indicates that the partial derivative is negative 87% of the weeks, and that the negative area comprises 97% of the total area. The partial derivative signals indicate that: **(a)** A brand’s promotions preyed on its own sales shares (an outcome documented in the literature); **(b)** A brand’s promotions generated the offsetting benefit of cannibalizing sales shares of competing brands; and **(c)** The sales shares of ABInbev brands (S1 and S3) were mutually-beneficial, but the sales share of S3 was preyed on by the outside brand owned by a different brewery (S9).

To quantify these interactions with S-mapping, we constructed an empirical attractor manifold with the variables S1, S1P, S3, S3P, and S9 serving as phase space coordinates. The computed partial derivatives quantifying each interaction over time are the black curves with areas shaded black between the curves and the zero-axis to highlight weeks when the interaction is positive or negative (Fig 13). Since computed partial derivatives are volatile time series, we applied singular spectrum analysis to isolate signals measuring systematic interactive behavior (shaded red).

The partial-derivative signals are mostly either positive or negative for the majority of weeks in the POR. We characterize this regularity and its importance with two measures: (1) the percentage of weeks that each partial-derivative signal is mostly positive/negative; and (2) the relative magnitude of positive/negative areas to total area between the curve and the zero-axis. We report these measures in the headings to each plot. For example, '∂S1/∂S1P (Negative: 0.87, 0.97)’ reports that this partial derivative is negative 87% of the weeks, and that the negative area comprises 97% of the total area (Fig 13A, leftward plot).

We first investigate the bilateral interaction between a brand’s promotions and its own sales shares (Fig 13A) for the case of the top selling brand configuration in the category (Leffe Blonde Lager Bottles 4X330 1320ml). We observe that ∂S1P/∂S1 was positive 71% of the weeks, indicating that an incremental increase in the configuration’s weekly market share (S1) most often marginally increased its decision to promote (S1P). The paired interaction, ∂S1/∂S1P, was negative 87% of the weeks, indicating that an incremental increase in promotions most often drove down marginal sales shares. Taking an ecological interpretation, the configuration’s promotions (S1P) preyed on its sales shares (S1). Turning to the third top selling brand configuration in the category (Hoegaarden White Lager Bottles 4X330 1320ml), we observe the same predatory behavior of promotions on own sales shares when examining the paired interactions, ∂S3P/∂S3 and ∂S3/∂S3P.

We next examine the impact of a brand’s promotions on the sales shares of competitors (Fig 13B). Considering first the ABInbev brands (Leffe and Hoegaarden), we observe that each brand’s promotions cannibalized the sales shares of the other in most weeks. Hoegaarden’s decision to promote (S3P) had a mostly negative marginal impact on Leffe’s sales shares (S1) since ∂S1/∂S3P was negative 73% of the weeks; while an incremental increase in Leffe’s sales shares marginally increased Hoegaarden’s promotions since ∂S3P/∂S1 was positive in 73% of the weeks. We observe even stronger evidence of similar cannibalistic behavior of Leffe promotions on Hoegaarden sales shares since ∂S3/∂S1P was negative 81% of the weeks and ∂S1P/∂S3 was positive 88% of the weeks. The latter partial derivative, ∂S1P/∂S3, tells us that, in most weeks, Leffe’s marginally increased its promotions (S1P) in response to an incremental increase in Hoegaarden’s sales share (S3). In striking constrast, the marginal response of Leffe’s promotions to an incremental increase in the sales share of non-ABInbev brand Innis & Gunn (the 9^th^ top selling brand in the category), ∂S1P/∂S9, was negative in 88% of the weeks (Fig 13B, rightward plot).

We further found that incremental increases in the sales shares of ABInbev brands Leffe (S1) and Hoegaarden (S3) marginally increased the sales shares of the other in most weeks (Fig 13C, leftward two plots). The marginal impact of Hoegaarden on top seller Leffe was especially strong since ∂S1/∂S3was positive 100% of the weeks. Ecologically speaking, the sales shares of ABInbev brands coevolved in a mutually-beneficial or *symbiotic* relationship. In another marked contrast, 9^th^-largest selling non-ABInbev brand Innis & Gunn tended to prey on the sales shares of 3^rd^-largest selling Hoegaarden (Fig 13C, rightward plots). An incremental increase in Innis & Gunn’s sales shares (S9) marginally decreased the sales share of Hoegaarden (S3) 77% of the weeks; while an incremental increase in Hoegaarden sales shares marginally increased Innis & Gunn’s sales shares about half of the weeks.

## Discussion

In making sense of these results, we emphasize that NLTS provides positive analysis of behavior that ‘actually happened’. Comparing this to what ‘should have happened’ had brands behaved ‘rationally’ in the judgement of the researcher may well be inaccurate without direct knowledge of their internal business objectives—like shooting an arrow without seeing the target. Moreover, brands realizing undesireable outcomes might have reasonably anticipated something better, and avoided the behavior in hindsight. We are limited to speculating a few of many possible explanations for why brands engaged in apparently puzzling behavior uncovered by NLTS diagnostics.

The first puzzling question is why brands would promote when, in most weeks, promotions preyed on their own market shares. One possibility is that brands reasonably anticipated the opposite outcome (i.e., that promotions would marginally increase their own market shares), but these expectations were not met possibly because promotions caused consumers to downgrade their perceptions of brand quality or lower their price expectations to the promotional level, and that these responses overwhelmed an increase in brand loyalty or an inertia repurchasing effect in most weeks. Another possibility is that brands invested in promotions to cannibalize the sales shares of close competitors, which turned out to be the case in most weeks even between brands owned by the same multinational brewery. This intra-brewery brand competition offers some empirical evidence that the international beer market was contestable despite being dominated by a few large multinational breweries [49].

But this begs the next puzzling question: Why did this intra-brewery brand competition exist given evidence that intra-brewery sales shares were strongly mutually beneficial? A post hoc rationalization for this perplexing behavior is not obvious. ABInbev might benefit by using this informtion to rethink the promotion strategies of its internal brands in the specialty beer market.

## Conclusion

In this paper, we applied a novel empirical approach—Nonlinear Time Series Analysis (NLTS)—to reconstruct real-world market dynamics concealed in volatile observed time-series records, and used this information to respond to key empirical questions raised in the literature regarding how price promotions and sales shares among competing brands systematically interact over time. We first tackled the essential preliminary question of whether these interactions are systematic in our dataset in the first place; in particular, whether the data conceal random interactions exogenously forced by linear-stochastic real-world market dynamics, or deterministic interactions endogenously forced by real-world nonlinear market dynamics. We found that market dynamics reconstructed from the Tesco dataset are largely deterministic, low-dimensional, and nonlinear.

Borrowing new methods from ecosystem dynamics used to investigate interspecies interactions, we next detected real-world interactions among the promotions and sales of brands, and characterized the nature of detected interactions over time by computing partial derivatives measuring the marginal response of one market variable to an incremental change in another. We uncovered evidence that a brand’s promotions preyed on its own sales shares (an outcome documented in the literature), but generated the offsetting benefit of cannibalizing sales shares of competing brands. We also found evidence that interactions between brands owned by the same multinational brewery differed from their interactions with outside brands. In particular, the sales shares of brands owned by the same brewery were mutually beneficial; whereas the sales shares of brands owned by different breweries preyed on each other’s market shares.

In general, NLTS opens a new window on analyzing complex time series records by illuminating otherwise concealed information: Is observed volatility generated by exogenous shocks to a self-correcting system or endogenous unstable dynamic behavior that is not self-correcting? This distinction—recognized increasingly as pivotal in managing and regulating real-world dynamic systems—is imperceptible to the naked eye, and to exploratory empirical approaches presuming stochastic forcing.

There are important caveats to applying NLTS, especially to short and noisy time-series records encountered in practice. First, we cannot reasonably expect to reconstruct the complex folding and fractal patterns of a real-world attractor [50]. We must lower our expectations to reconstruct a sampling or skeleton of the real-world attractor [51]. Second, these methods can fall short of reconstructing even a skeleton attractor for several reasons. Most obviously, a low-dimensional nonlinear real-world attractor may not exist. Or, the data may be insufficiently informative to reconstruct the real-world attractor even if it does exist. For example, the time series might only sample transitory dynamics heading toward the attractor. However, we do not know any of this until we have tested the data for it.

## Supporting information

S1 File Raw sales data. This spreadsheed contains raw data on sales of specialty beer brands provided by scanned purchases of Tesco ClubCard in the UK. These data are available to subscribers by dunnhumby (a subsidiary of Tesco) via a web-portal. Coauthor Andrew Fearne was provided original access to these data pursuant to a longstanding research relationship with dunnhumby.
(XLSX)

S2 File Raw promotions data. This spreadsheet contains raw data on promotions of specialty beer brands collected by dunnhumby, which makes these data available to subscribers via a web-portal. Coauthor Andrew Fearne was provided original access to these data pursuant to a longstanding research relationship with dunnhumby.
(XLS)

S3 File Promotions put into equivalent monetary value. Promotions were reported in S2 File as savings to consumers: S = PR—PP, where PR is the regular (pre-promotion) unit price and PP is the promotional counterpart. Savings were reported directly for simple price cuts. For more sophisticated promotions—such as *any two for £3*—the ‘equivalent single promotional price’ was reported (PP = *£3/2 = £1*.*50*), along with the ‘depth of cut’ (*DC*) measuring the percentage decrease from the regular price (for example, 3.85%). In S3 File, we computed consumer savings for these promotions indirectly by solving for the unreported regular price from the definition of DC: DC = (PR—PP)/PR -> PR = PP/(1—DC), and substituting it into the consumer savings equation: S = PP/(1—DC)—PP = *£1*.*50(0*.*04) = £0*.*06*.
(XLS)
